# Thermosensitive Polyhedral Oligomeric Silsesquioxane Hybrid Hydrogel Enhances the Antibacterial Efficiency of Erythromycin in Bacterial Keratitis

**DOI:** 10.34133/bmr.0033

**Published:** 2024-07-22

**Authors:** Lan Zheng, Ying Chen, Yi Han, Jingwei Lin, Kai Fan, Mengyuan Wang, Ting Teng, Xiuqin Yang, Lingjie Ke, Muyuan Li, Shujia Guo, Zibiao Li, Yunlong Wu, Cheng Li

**Affiliations:** ^1^ Fujian Provincial Key Laboratory of Ophthalmology and Visual Science & Ocular Surface and Corneal Diseases, Eye Institute & Affiliated Xiamen Eye Center & Affiliated First Hospital, School of Medicine, Xiamen University, Xiamen 361102, PR China.; ^2^ Fujian Provincial Key Laboratory of Innovative Drug Target Research and State Key Laboratory of Cellular Stress Biology, School of Pharmaceutical Sciences, Xiamen University, Xiamen 361102, PR China.; ^3^ Department of Ophthalmology, The First Affiliated Hospital of University of South China, Hengyang Medical School, University of South China, Hengyang, Hunan, 421001, PR China.; ^4^ Shandong First Medical University & Shandong Academy of Medical Sciences, Jinan 250117,Shandong Province, PR China.; ^5^ Institute of Materials Research and Engineering, A*STAR (Agency for Science, Technology and Research), Singapore138634, Singapore.; ^6^ Huaxia Eye Hospital of Quanzhou, Quanzhou, Fujian 362000, China.

## Abstract

Bacterial keratitis is a serious ocular infection that can impair vision or even cause blindness. The clinical use of antibiotics is limited due to their low bioavailability and drug resistance. Hence, there is a need to develop a novel drug delivery system for this infectious disease. In this study, erythromycin (EM) was encapsulated into a bifunctional polyhedral oligomeric silsesquioxane (BPOSS) with the backbone of the poly-PEG/PPG urethane (BPEP) hydrogel with the aim of improving the drug efficiency in treating bacterial keratitis. A comprehensive characterization of the BPEP hydrogel was performed, and its biocompatibility was assessed. Furthermore, we carried out the evaluation of the antimicrobial effect of the BPEP-EM hydrogel in *S. aureus* keratitis using in vivo mouse model. The BPEP hydrogel exhibited self-assembling and thermogelling properties, which assisted the drug loading of drug EM and improved its water solubility. Furthermore, the BPEP hydrogel could effectively bind with mucin on the ocular surface, thereby markedly prolonging the ocular residence time of EM. In vivo testing confirmed that the BPEP-EM hydrogel exerted a potent therapeutic action in the mouse model of bacterial keratitis. In addition, the hydrogel also exhibited an excellent biocompatibility. Our findings demonstrate that the BPEP-EM hydrogel showed a superior therapeutic effect in bacterial keratitis and demonstrated its potential as an ophthalmic formulation.

## Introduction

Bacterial keratitis (BK) is a common and serious infection of the cornea caused by bacteria that invade and multiply in the eye. It is the worldwide leading cause of microbial keratitis and the common kind of infectious keratitis [1–5]. *Staphylococcus aureus* and *Pseudomonas aeruginosa* are most common pathogens leading to BK [6]. The bacterial invasion due to these pathogens damages the corneal epithelium, causes stromal inflammation and ulceration, and may lead to irreversible vision loss [7,8]. According to incomplete statistics, BK carries the highest risk for vision impairment and blindness globally, particularly in industrial workers and those wearing contact lens [9,10]. The standard treatment for BK is topical antibiotics, with fluoroquinolones being the preferred choice. However, the widespread use of fluoroquinolones can induce drug resistance, which reduces their effectiveness [11–13]. In such cases, the antimicrobial peptides can serve as natural antibiotics by exhibiting a broad-spectrum resistance to a variety of pathogens including bacteria. However, their application is constrained by their limited bioactivity and potential for biological toxicity [14–16].

Erythromycin (EM) is a macrolide antibiotic that is active against the majority of Gram-positive bacteria and is used to treat a variety of bacteria-induced diseases [17,18]. Unfortunately, EM has poor solubility in water-based vehicles, which limits the formulation of the drug in eye drops [19]. Furthermore, the eyeball has a unique physiological structure in which the corneal epithelial barrier and tear film severely limit medication absorption into the ocular surface, resulting in limited bioavailability and repeated instillation. However, repeated instillation may cause major adverse effects [20]. Thus, it is critical to enhance the residence duration of the medication on the ocular surface and tackle the problem of inadequate drug solubility [21,22]. Prior research has explored the use of EM encapsulated in polymer micelles for an innovative eye drop formulation, aimed at enhancing the bioavailability of drug. However, the efficacy of this approach has yet to be confirmed through in vivo validation [23].

Currently, strategies have been designed to address the problem of ocular drug delivery of pharmacological agents by improving the bioavailability. These strategies include adding solubility enhancers to increase the drug solubility in the formulation, adding penetration enhancers to facilitate the drug passage through the corneal epithelium, and using long-acting drug delivery systems based on adhesive polymers such as hydrogels, nanomicelles, liposomes, microneedles, and dendrimers [24–31]. Hydrogel drug delivery technologies have attracted a lot of interest in recent years [32].

Hydrogels have been widely used as soft materials for biomedical applications such as drug delivery, tissue engineering, wound dressing, bioimaging, and biosensors [33–36]. Due to the intrinsic physically or chemically crosslinked polymeric networks, hydrogels are able to absorb large amount of water without dissolution and generate similar physicochemical properties as to the natural tissues or organs [37]. Particularly, over the past few decades, much efforts have been devoted in the development of “smart hydrogels” with stimuli responsiveness to the environmental stimuli such as temperature, pH, and light, where any change in these factors can relatively cause large changes in terms of network structure, swelling behavior, mechanical strength, or permeability [38].

Thermogels present a temperature-sensitive subset of hydrogels, with characteristic reversible sol–gel transition upon heating/cooling [39]. Such unique temperature responsiveness enables them to be utilized as injectable materials and administered in a minimally invasive way. The resultant hydrogels not only adapt well to the irregular shape of the implantation site but could be loaded with diverse therapeutic agents to serve their intended purpose [40]. Currently, several thermal-sensitive polymers including poly(N-isopropylacrylamide), polypeptides, block copolymers of poly(ethylene glycol) (PEG) and poly(propylene glycol) (PPG), and block copolymers of PEG and polyesters (such as Pluronic F127) are main systems of study [41,42]. Among them, our group has developed a versatile strategy to create thermogelling systems based on polyurethane of PEG, PPG, and a third hydrophobic component, with the advantage of tailorable critical gelation temperature (CGT), low critical gelation concentration (CGC), high resistance to hydrolysis, and other functionality rendered by the third component [43,44]. For example, polylactic acid, polycaprolactone, polyhydroxyalkanoates, and polycarbonates in copolymers can provide the desirable biodegradability [45–48], tetraphenylethene in copolymers shows strong aggregation-induced emission effect [49], and spiropyran in copolymers can give rise to light responsiveness in sol–gel transition [50]. These materials with excellent properties have an immense potential for various applications in drug delivery, vitreous substitute, bioimaging, and sensors [49–52].

**Fig. 1.**
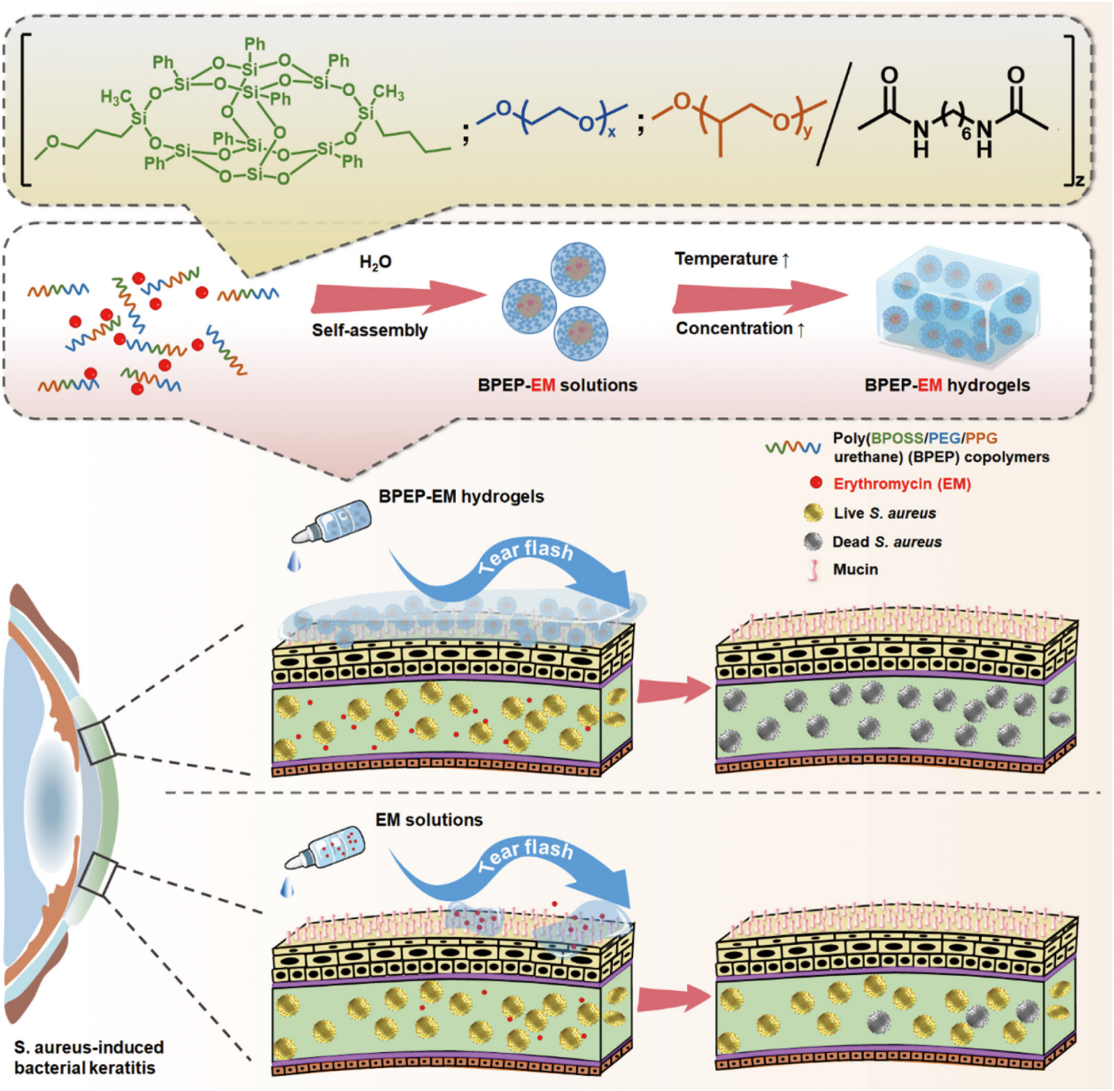
Schematic representation of the synthesis of BPEP-EM hydrogels for the therapy of bacterial keratitis.

In this study, we introduced a bifunctional polyhedral oligomeric silsesquioxane (BPOSS) to the backbone of poly-PEG/PPG urethane (BPEP) in order to obtain a novel kind of inorganic/organic hybrid copolymers with thermogelling properties. The copolymers were systematically studied for the molecular characteristics, thermal properties, micellization in dilute aqueous solution, and gelation in concentrated aqueous solution. The developed inorganic/organic hybrid copolymer could bind to the ocular surface mucin and facilitated the loading of the insoluble drug EM by increasing its water solubility and residence time on the ocular surface. Subsequently, an in vivo mouse model and cell tests were employed to explore the therapeutic impact and biological safety in treating *S. aureus* keratitis (Fig. 1). The results reveal that the hydrogel was biocompatible and could greatly increase the drug medication residence duration at the ocular surface. Furthermore, the BPOSS-PEG-PPG-EM (BPEP-EM) hydrogel exhibited a better therapeutic effect on BK than EM solution and Pluronic F127-EM (F127-EM), which makes it a promising candidate for treating corneal infections.

## Materials and Methods

### Materials

Methyltrichlorosilane (97%), allyloxytrimethyl-silane (98%), dichloromethylsilane (98%), Karstedt catalyst (96%), and phenyltrimethoxysilane (98%) were supplied by Alfa Aesar (Shanghai, China). Triethylamine (99%), sodium hydroxide (98%), isopropanol (99%), and dichloromethane (99.5%) were provided by Aladdin Reagent (Shanghai, China). Methanol, toluene, and hexane were supplied by Sinopharm Chemical Reagent Co. Ltd. (Shanghai, China). Anhydrous toluene, diethyl ether, hexane, methanol, and tetrahydrofuran (THF) were obtained from Acros Organics (Singapore). PEG (*M*_n_ = 2,000), PPG (*M*_n_ = 2,050), dibutyltin dilaurate (95%), 1,6-hexamethylene diisocyanate (98%), 1,6-diphenyl-1,3,5-hexatriene (98%), and deuterated chloroform (99.8 atom % D) were supplied by Sigma-Aldrich (Singapore). Dialysis tubing (molecular weight cutoff, 3,500 Da) was provided by Spectrum Laboratories (USA).

Dulbecco’s modified Eagle’s medium/Nutrient Mixture F-12 (DMEM/F-12) were obtained from HyClone (USA). Fetal bovine serum (FBS) was purchased from Gibco, and recombinant human epidermal growth factor (hEGF) (C209) was bought from Novoprotein. Phosphate-buffered saline (PBS) (1×) was purchased from Shanghai Yuanpei Biotechnology Co. Ltd. Penicillin–streptomycin solution, trypsin/0.25% EDTA, and insulin (human recombinant) were obtained from Shanghai Yeasen Technology Co. Ltd. Live-Dead Cell Staining Kit (catalog K2081) was bought from APExBIO Technology (Shanghai, China), and 4′,6-diamidino-2-phenylindole (DAPI) (catalog H-1200) was provided by Vector (Burlingame, CA, USA). CM5 sensor chip and Amine coupling kit were bought from GE Healthcare (Chicago, IL, USA).

Carbomer eye gel bought was obtained from Bausch Lomb (Rochester, NY, USA), and Carbomer eye drops were purchased from Bausch Lomb. Optimal cutting temperature (OCT) compound (Tissue-Tek OCT) was provided by Sakura Finetek (Torrance, CA, USA). Paraformaldehyde (4%) and the hematoxylin and eosin (H&E) staining kit were obtained from Biosharp (Anhui, China). The Masson’s Trichrome staining kit was purchased from Jiancheng Bioengineering Institute (Nanjing, China). The anti-P63 antibody (1:200; catalog ab124762) and the anti-fibronectin antibody (1:200; catalog ab2413) were provided by Abcam Inc. (Cambridge, MA, USA). The anti-CD11a antibody (1:200; catalog ab52895) was provided by Abcam Inc. (Cambridge, MA, USA). Alexa Fluor 488 Phalloidins (1:200; catalog A-12379), Alexa Fluor 594 Phalloidins (1:200; catalog A-12381), and donkey anti-rabbit Alexa Fluor 488-conjugated immunoglobulin G (IgG) (1:300; catalog A-21206) were provided by Invitrogen (Eugene, OR, USA).

### Animals

Female C57BL/6 mice, weighing 15 to 25 g and aged 6 to 10 weeks, were acquired from the Animal Research Center of Xiamen University (Xiamen, China). The mice were housed under standard conditions (20 to 25 °C, 50 to 70% relative humidity, with a 12-h light/dark cycle, ambient noise below 60 dB) and had ad libitum access to standard chow and water. The research protocol was approved by the Xiamen University Experimental Animal Ethics Committee. Additionally, the study adhered to the guidelines set forth in the Association for Research in Vision and Ophthalmology,s Statement for the Use of Animals in Ophthalmic and Vision Research.

### Synthesis of BPOSS and poly(BPOSS/PEG/PPG urethane) (BPEP) copolymers

A four-step synthetic route that included hydrolytic polycondensation, silylation reaction, hydrosilylation reaction, and deprotection reaction was adopted to obtain BPOSS as per our previous work [53]. Initially, phenyltrimethoxysilane (30 ml, 0.16 mol), sodium hydroxide (4.26 g, 0.1 mol), deionized water (3.24 g, 0.18 mol), and isopropyl alcohol (160 ml) were taken into a 250-ml three-port flask under nitrogen atmosphere and stirred at 96 °C reflux for 4 h and then at room temperature for 15 h. After rotating evaporation and vacuum drying (60 °C) for 12 h, 22.9 g of product 1 was obtained. Subsequently, product 1 (22.48 g, 19.4 mmol), triethylamine (5.89 g, 58.2 mmol), and THF (100 ml) were subjected into a 250-ml round-bottom flask and cooled in an ice water bath, to which 10 ml of dichloromethyl silane THF solution (6.69 g, 58.2 mmol) was added in a nitrogen atmosphere. The reactants were successively stirred at 0 °C for 1 h and then at 70 °C for 3 h. After centrifugation, rotary evaporation, washing with methanol (100 ml) three times, and vacuum drying (40 °C) for 24 h, 9.2 g of product 2 was obtained. Next, product 2 (5.44 g, 4.7 mmol), allyloxy-trimethylsilane (3.67 g, 28.2 mmol), and toluene (50 ml) were put into 100-ml round-bottom flares and treated with nitrogen gas for three exhaust refills. Subsequently, Karstedt catalyst was added and the reaction mixture was stirred at 95 °C for 36 h, rotary evaporated, and vacuum dried (40 °C) for 24 h, to 5.8 g of product. In the last step, product 3 (3.0 g, 2.1 mmol), methanol (90 ml), and dichloromethane (90 ml) were taken into a 250-ml round-bottom flask, stirred under nitrogen atmosphere, and stirred with a dropwise addition of 10 ml of methanolic solution of methyltrichlorosilane (0.94 g, 6.3 mmol). After stirring at room temperature for 5 h, the final product BPOSS was obtained by rotary evaporation, THF-hexane (1:1, v/v) washing, and vacuum drying (40 °C) for 24 h.

The synthesis of poly(BPOSS/PEG/PPG urethane) was carried out via a similar method reported in our previous work [54]. PEG and PPG were copolymerized with BPOSS to yield BPEP copolymer using chain extender 1,6-diphenyl-1,3,5-hexatriene and catalyst dibutyltin dilaurate. The ratio of PEG and PPG was fixed at 2:1, and the content of BPOSS was 0.5, 1, and 2 wt %, respectively; hence, the copolymers were named 0.5BPEP, 1BPEP, and 2BPEP (BP representing BPOSS, E representing PEG, and P representing PPG). PEG, PPG, and BPOSS were mixed in appropriate proportions and added to a 250-ml round-bottom flask with anhydrous toluene (100 ml). Most toluene and traces of water were removed by rotary evaporation (repeated twice). When 10 ml of toluene remained, the mixture was stirred at 110 °C, and 1,6-hexamethylene diisocyanate (0.84 ml, 5.2 mmol) and two drops of dibutyltin dilaurate were added in an argon atmosphere. When the reaction became obviously viscous, another 10 ml of anhydrous toluene was added. After the reaction for 24 h, 1BPEP and 2BPEP were obtained after precipitating with n-hexane-ethyl ether (3:7, v/v), resolubilizing in isopropyl alcohol and deionized water dialysis, and freeze-drying.

### Synthesis of BPEP hydrogel loaded with EM

Preparation of BPEP-EM: 5 mg of EM and 40 mg of 1BPEP/2BPEP were dissolved in 100 and 200 μl of ethanol, respectively, and then mixed. The mixed droplets were added to 1 ml of ddH_2_O, ultrasonicated for 15 min, and subjected to nitrogen blowing for 15 min, and ddH_2_O was added to keep the total volume of solution 1 ml. Subsequently, 80 mg of 1BPEP/2BPEP was added to the above solution to give a final concentration of BPEP/2BPEP of 12 wt %.

Preparation of Pluronic F127-EM (F127-EM): The preparation method was the same as that of BPEP-EM, but the final concentration of F127 was 24%.

### Dynamic light scattering measurement

DLS measurements were conducted for aqueous BPEP solutions to reveal the particle size and size distribution at different concentrations (0.1, 0.5, and 1 wt %) and temperatures (25, 37, and 70 °C). All the measurements were performed using a Malvern Nano ZetaSizer System at 633-nm laser light and 173° scattering angle.

### Sol–gel transition behaviors

Three BPEP copolymers were dissolved in water with the concentration varying from 2 to 20 wt %. The samples were cooled at 4 °C for 1 day to achieve complete dissolution. The state of the samples at different temperature (2 to 82 °C) was recorded at an interval of 2 °C. The gel state was distinguished from the sol state by ascertaining whether the samples would flow after inverting the vials.

### Rheological analysis

Temperature ramps from 20 to 37 °C and temperature sweeps from 4 to 80 °C at a rate of 5 °C min^−1^ for the samples were measured on a TA Instruments DHR-3 rheometer equipped with a parallel plate. The oscillation amplitude was fixed at 1%, and the oscillation frequency was fixed at 1 Hz.

### Lower critical solution temperature determination

Three BPEP copolymers were dissolved in water at the concentration of 2 wt % for ultraviolet–visible (UV–vis) spectroscopy on the Shimadzu UV-2501 PC spectrophotometer equipped with a temperature control module (25 to 75 °C). UV–vis spectra for the samples were recorded at an interval of 1 °C.

### Light transmittance and scanning electron microscopy detection of hydrogels

In order to compare the light transmittance of different hydrogel, commercially available EM oculentum and Pluronic F127 (F127) hydrogels were selected as controls. Specifically, 100 μl of 1BPEP-EM, 2BPEP-EM, and F127-EM was prepared and transferred to 96-well plates and then gelled at 34.5 °C. UV absorption values in the wavelength range of 390 to 780 nm were detected by a microplate reader (Molecular Devices, USA), and the light transmittance was calculated according to the calculation formula: (Absorption value, *A*) = −Log (Light transmittance, *T*) %.

The scanning electron microscope (SEM; SUPRA55 SAPPHIRE Scanning Electron Microscope, Zeiss, Germany) was used to analyze the microscopic morphology of the hydrogel. Following the freeze-drying of the 2BPEP and 2BPEP-EM hydrogel, a portion of them were subjected to gold spraying, and photographs were captured.

### Degradation performance analysis of hydrogels

F127, 1BPEP, and 2BPEP hydrogels were prepared in 2-ml centrifuge tube and gelled at 34.5 °C. An equal volume of 1× PBS buffer solution was added to the upper part of the hydrogels, and the degradation ability of the hydrogels was analyzed at 34.5 °C and 50 rpm in a table concentrator. In order to regularly detect the degradation, the supernatants were removed and weighed at multiple time points, and the degradation capacity of different hydrogel systems was compared by monitoring the quality of the remaining hydrogel in the tube.

### Evaluation of sustained release of drug from the hydrogel system

1BPEP-EM, 2BPEP-EM, and F127-EM hydrogels were prepared in 2-ml centrifuge tube and gelled at 34.5 °C. In order to compare the sustained release ability of various hydrogels, 1× PBS buffer containing 0.2% Tween 20 (pH 7.4) was selected as the release medium, and the release medium was added to the upper end of the hydrogels. The experiment was performed at 34.5 °C and 100 rpm. Supernatants (500 μl) were collected at 0, 0.25, 0.5, 1, 2, 3, 4, 5, 6, 7, and 8 days, respectively, and the released doxorubicin contents were detected by high-performance liquid chromatography.

### Comparison of surface plasmon resonance properties of polymers

In order to further explore the adhesion of polymers, Biacore-T200 biomolecular interaction analyzer (GE, USA) was used to explore the interaction between the various polymers and mucin. In detail, we first removed the salt content by dialysis in ice 1× PBS buffer, and then freeze-dried mucin samples without salt were recovered. We immobilized mucin in the CM5 sensor chip by the amino coupling method, resulting in the final mucin immobilization level of 10,000 RU on the chip. We diluted 1BPEP, 2BPEP, and F127 with 1× PBS buffer to 3.906, 7.813, 1.563, 31.25, and 62.5 μM, respectively, and the following parameters were set to explore the protein–polymer interaction conditions: flow rate, 30 μl/min; contact time, 120 s; and separation time, 450. The interaction ability between various polymers and mucins was analyzed by Biacore-T200 evaluation software (2.0), and the kinetic constant (*K*_D_ value) was calculated by curve fitting of 1:1 binding model.

### In vitro biocompatibility

An immortalized human corneal epithelial (HCE) cell line was bought from the American Type Culture Collection (ATCC) and cultured in DMEM/F12 medium supplemented with 6% FBS, 1% penicillin–streptomycin solution, hEGF, and insulin at 37 °C, 5% CO_2_.

The methyl thiazolyl tetrazolium (MTT) assay was used to determine the cell viability. HCE cells were seeded in 96-well plates, and after 24 h, the following materials were introduced for toxicity testing: 1BPEP, 2BPEP, F127, 1BPEP-EM, 2BPEP-EM, F127-EM, and EM. The drug solution was withdrawn and replaced with MTT solution after 24 h of cocultivation, and the culture was maintained for another 4 h. After 4 h, the methazine crystals were dissolved with dimethyl sulfoxide (DMSO), and the absorbance was measured at 492 nm using a SpectraMax absorbance meter.

Additionally, the cells were planted on a 48-well plate and treated for 24 h with an 80 μg/ml EM solution. The development and morphology of the cells were monitored using an Axio Observer microscope, and the cells were cultivated continuously for 120 h for observation.

For LIVE/DEAD staining, HCE cells were grown on 48-well plates for 24 h before adding 80 μg/ml of the drug solution. After 24, 48, and 72 h of cocultivation, the cells were washed with PBS buffer and stained for 5 min with propidium iodide (PI) staining solution. After 5 min, the cells were rinsed with PBS buffer and stained for 15 min with calcein AM staining solution. Cells were rinsed with PBS buffer after 15 min and examined under a fluorescence microscope (Leica).

For F-actin/DAPI staining, HCE cells were cultivated in 48-well plates for 24 h before being treated with 80 μg/ml of drug solution and cultured for another 24, 48, and 72 h. Cells were fixed with 4% paraformaldehyde, permeabilized with 0.2% Triton, and then blocked with 2% FBS. Cells were treated with phalloidin for 1 h at room temperature before being stained with DAPI. For P63/DAPI staining, cells were blocked overnight with an anti-P63 antibody at 4 °C. The next day, cells were treated for 1 h with anti-rabbit Alexa Fluor 488-labeled IgG and mounted with DAPI. The images were captured using a fluorescent microscope (Leica, Wetzlar, Germany).

### In vitro antibacterial activity

The in vitro antibacterial activity was determined by the zone inhibition method. The culture medium required for *S. aureus* was sterilized by autoclaving at 121 °C for 15 min. Initially, *S. aureus* was inoculated into tryptone soy broth for liquid culture and incubated at 35 °C for 24 h. Subsequently, an *S. aureus* suspension with a density of 1 × 10^8^ colony-forming units (CFU)/ml was introduced onto agarose tryptone soy broth medium for solid culture. The sterilized filter paper was sliced into 6-mm discs and meticulously positioned on a petri dish. Five microliters of the pharmaceutical agent was administered onto the filter paper, ensuring that the concentration is identical in both the control and drug-infused solutions. After 24 h of incubation, the diameter of the inhibition zone was measured.

### Retention test of hydrogel on the ocular surface

1BPEP-EM, 2BPEP-EM, F127-EM, and EM were topically administered on the ocular surface of C57BL/6 mice, and pictures were taken with a slit lamp after blinking.

### Establishment and treatment of BK in mice

A model of BK was established by injecting *S. aureus* into the corneal stroma of the mouse. In a nutshell, C57BL/6 female mice (6 to 8 weeks) were injected with 1% pentobarbital for anesthesia, a 32-gauge needle was used to establish a deep tunnel through the corneal stroma, and a 33-gauge needle was used to inject 2 μl of *S. aureus* suspension with a concentration of 5 × 10^7^ CFU/ml (washed three times with PBS). Different eye drops were applied twice a day for medication therapy after 24 h. At the end of experiments, the mice were sacrificed, and the eyes were collected and embedded in OCT compound. The study groups consisted of untreated mice with BK (MODEL) and those treated with 1BPEP, 2BPEP, F127, PBS-EM, F127-EM, 1BPEP-EM, and 2BPEP-EM, respectively.

### Observation of BK in mice

Slit lamp, optical coherence tomography (OCT), and intravital confocal microscopy were used to examine the infected mice on the first, third, fifth, and seventh days following infection. Slit lamp observations of ocular surface symptoms in mice were rated using previously published literature, with slight adjustments [55] (Table 1). An intravital confocal microscope was used to make the full-thickness scans of the mouse corneas (Heidelberg Engineering, Heidelberg, Germany). The sedated mice were placed on the platform with carbomer eye drop invading solution, and their corneas were positioned with the objective lens for scanning. The OPTOPROBE system was used to capture the OCT pictures of the mouse corneas. In a nutshell, sedated mice were put on a rack and their corneas were scanned. The thickness of the cornea was measured using Matlab-based analytical tools.

**Table 1.** Criteria for symptoms of the murine corneas

	Grade 0	Grade 1	Grade 2	Grade 3	Grade 4	Grade 5	Grade 6
Area of corneal opacity	0	1–15%	16–30%	31–45%	46–60%	61–75%	76–100%
Density of corneal opacity	Not cloudy	Slight cloudiness, structure of iris and pupil discernible	Slight cloudiness, structure of iris discernible	Cloudy, partial iris discernible	Moderate cloudiness, iris invisible	Cloudy, opacity not uniform	Uniform opacity

### Histology and immunofluorescence staining

The eyeballs were embedded with OCT compound and cut into 6-μm thickness sections for staining. For H&E staining, tissue sections were immersed in 4% paraformaldehyde, followed by a 5-min hematoxylin staining and subsequent rinsing. They are then stained with eosin for 2 min, rinsed, dehydrated through a series of xylene and alcohol washes, and finally mounted with neutral resin (H-5000). For Masson staining, after initial fixation in 4% paraformaldehyde, the sections were stained using a trichrome staining kit. Finally, photographs were taken and examined under a light microscope (Eclipse E400 with DS-Fi1, Nikon, Melville, NY, USA).

For immunofluorescence staining, fixation with 4% paraformaldehyde was initially employed. Triton X-100 (0.2%) permeabilized the tissue slices, which were then blocked with 2% bovine serum albumin and incubated at 4 °C overnight with the monoclonal anti-fibronectin and polyclonal anti-CD11a antibodies. The following day, the sections were incubated with donkey anti-rabbit Alexa Fluor 488-conjugated IgG for 1 h at room temperature, mounted with DAPI, and visualized under a fluorescence microscope (Leica DM2500).

### Safety testing

C57BL/6 female mice (6 to 8 weeks) were continuously administered with eye drops for 7 and 20 days. The eye drops included EM, F127-EM, 1BPEP-EM, and 2BPEP-EM. OCT and fundus images of mice were collected after 7 and 20 days by using an OPTOPROBE system (China). Mice were sedated with 1% pentobarbital, and their pupils were dilated with tropicamide phenylephrine eye drops before collecting the OCT and fundus pictures of the eyes. The mice were given 10% sodium fluorescein intraperitoneally, and fundus fluorescein angiography was performed to examine the fundus.

### Statistical analysis

All data were expressed as the mean ± SEM. Statistical significance was determined through one-way analysis of variance (ANOVA) or the unpaired Student’s *t* test (*P < 0.05, **P < 0.01, ***P < 0.001), in accordance with the data,s normal distribution, utilizing GraphPad Prism 8.0 software (GraphPad Software, San Diego, CA, USA). *P* < 0.05 was used to determine the statistical significance.

## Results and Discussion

### Preparation and characterization of micelles based on BPEP copolymers

BPEP copolymers comprise of hydrophilic PEG, hydrophobic BPOSS, and PPG endowed with reversible temperature-dependent hydrophilic/hydrophobic transition, which prompts them to form micelles in aqueous solution above a certain concentration and respond to temperature changes (Fig. S1 and Table S1). Typically, the hydrophobic moieties were condensed in the core of BPEP micelles by hydrophobic interactions and wrapped by hydrophilic moieties to form stable molecular assemblies.

**Fig. 2.**
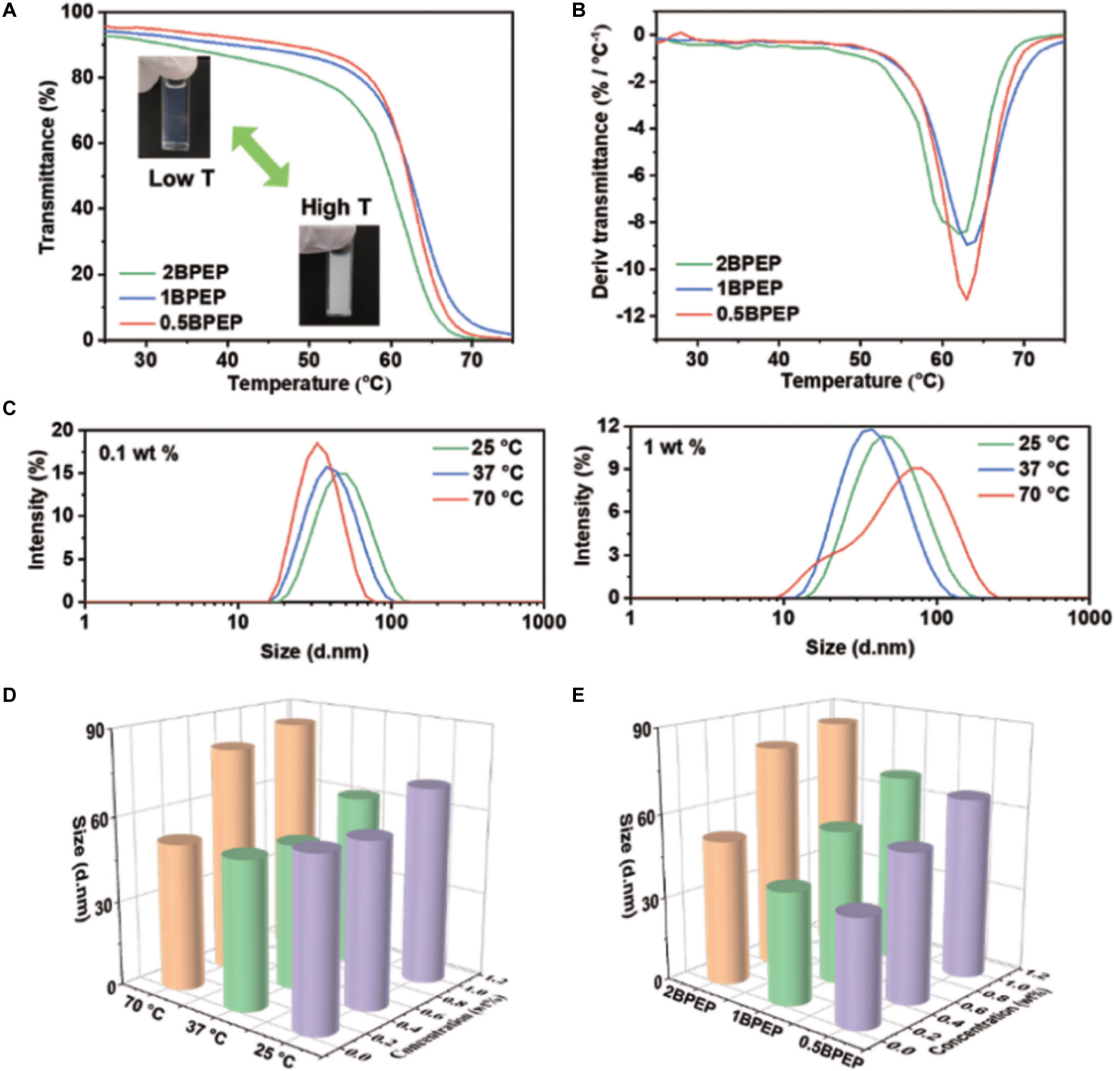
(A) Transmittance of aqueous BPEP solutions (2 wt %) in temperature range of 25 to 75 °C. (B) LCST determination from the derivative plots of (A). (C) Size distribution (by intensity) of micelles in aqueous 0.5BPEP solution at different concentration and temperature. (D) Mean particle size related to temperature and concentration for 2BPEP. (E) Mean particle size related to BPEP type and concentration at 70 °C.

On account of the enhanced hydrophobicity in BPEP copolymers, with an increase in the temperature, BPEP micelles are apt to shrink and aggregate to regain the hydrophilic/hydrophobic balance, leading to a growth in the size of assemblies. As a result, the aqueous BPEP solution would become more turbid upon heating, and the transmittance reduction from nearly 100% to 0% would be completed in a narrow temperature range (Fig. 2A). For three BPEP copolymers with concentration fixed at 2 wt %, we found that samples with higher BPOSS content showed a rapid decrease of transmittance benefitting from the strong hydrophobicity and self-assembly capacity of BPOSS (Fig. 2B). However, their lower critical solution temperature (LCST) values, which were defined as the temperature with the maximum descending rate, were very close in the range of 62 to 63 °C, probably because the BPOSS content was too low to make a big difference.

Next, we studied the influence of the factors including concentration, temperature, and BPOSS content on the particle size of BPEP in water by dynamic light scattering (DLS) and obtained their mean diameters (Table S1). At a concentration of 0.1 wt %, all three BPEP micelles presented a monotonic decrease in the mean particle size and a narrowing of size distribution with an increase in the temperature (Fig. 2C and D). On the contrary, at a concentration of 0.5 and 1 wt %, the mean particle size initially decreased and later increased, and the size distribution also narrowed first and later broadened, suggesting a favorable aggregation and rearrangement of micelles at higher concentration. In terms of BPOSS content, 0.5BPEP and 1BPEP showed a similar particle size at fixed temperature and concentration, while the particle size for 2BPEP was much higher (Fig. 2E), offering a helpful guidance for their applications in in vivo drug vehicles.

### Sol–gel phase transition of BPEP copolymers

**Fig. 3.**
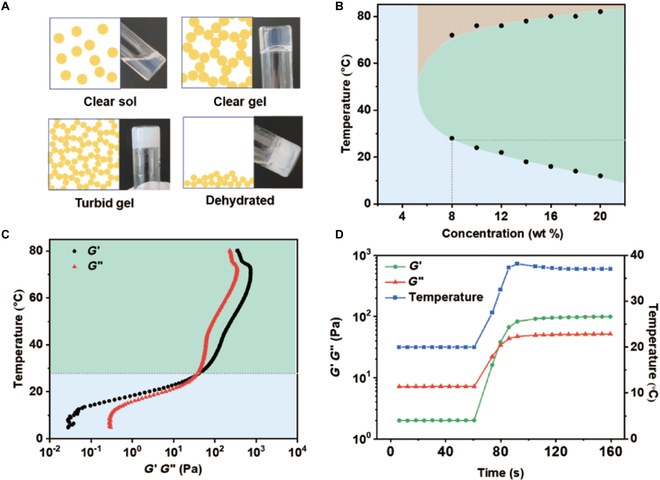
(A) Illustration for possible mechanism of phase transition in aqueous BPEP solution. (B) Phase diagram of 1BPEP determined by tube inverting method. (C) Rheological properties of 1BPEP (8 wt %) sample in temperature sweep. (D) Rheological properties of 1BPEP (8 wt %) sample in time ramp.

When the concentration and temperature of aqueous BPEP solution were both high enough, micelle aggregation and packing were favorable, generating micellar networks on a microlevel and gelation on a macrolevel (Fig. 3A). In addition, variation of micelle size triggered by temperature change was deemed to have an impact on the mesh size of their micelle networks, reflecting in the transformation of clear gel to turbid gel. At higher temperature, the micellar networks collapsed due to imbalanced hydrophilicity/hydrophobicity and exhibited dehydration and precipitation.

The concentration/temperature-dependent phase transition of BPEP copolymers was characterized by tube inverting method in the range of 2 to 82 °C, and the resultant phase diagrams are shown in Fig. 3B and Fig. S2A and B. The estimated CGC was as low as 5 wt % and most of the CGT corresponding to given concentration fell in the physiological temperature range. Further, their thermal-sensitive properties were investigated by rheological tests, where the sol state was regarded to have storage modulus *G*′ smaller than the loss modulus *G*″, while the gel state was the opposite. A distinct sol–gel transition was observed in the temperature sweep graphs for these BPEP copolymers (Fig. 3C and Fig. S2C and D), with a gelation temperature of 32 °C for 0.5BPEP (10 wt %), 28 °C for 1BPEP (8 wt %), and 24 for 2BPEP (6 wt %), all well coinciding with the CGT determined from phase diagrams. At the higher temperature zone, *G*′ and *G*″ were close to each other, again leading to the dehydration of gel state. In contrast, a fast sol–gel transition rate with a time frame of only seconds was observed in response to the temperature change from the temperature ramp graphs between 20 and 37 °C (Fig. 3D and Fig. S2E and F). These results indicated good thermogelling properties of BPEP copolymers and can offer a great potential in biomedical applications.

### Light transmittance and microstructure of hydrogels

**Fig. 4.**
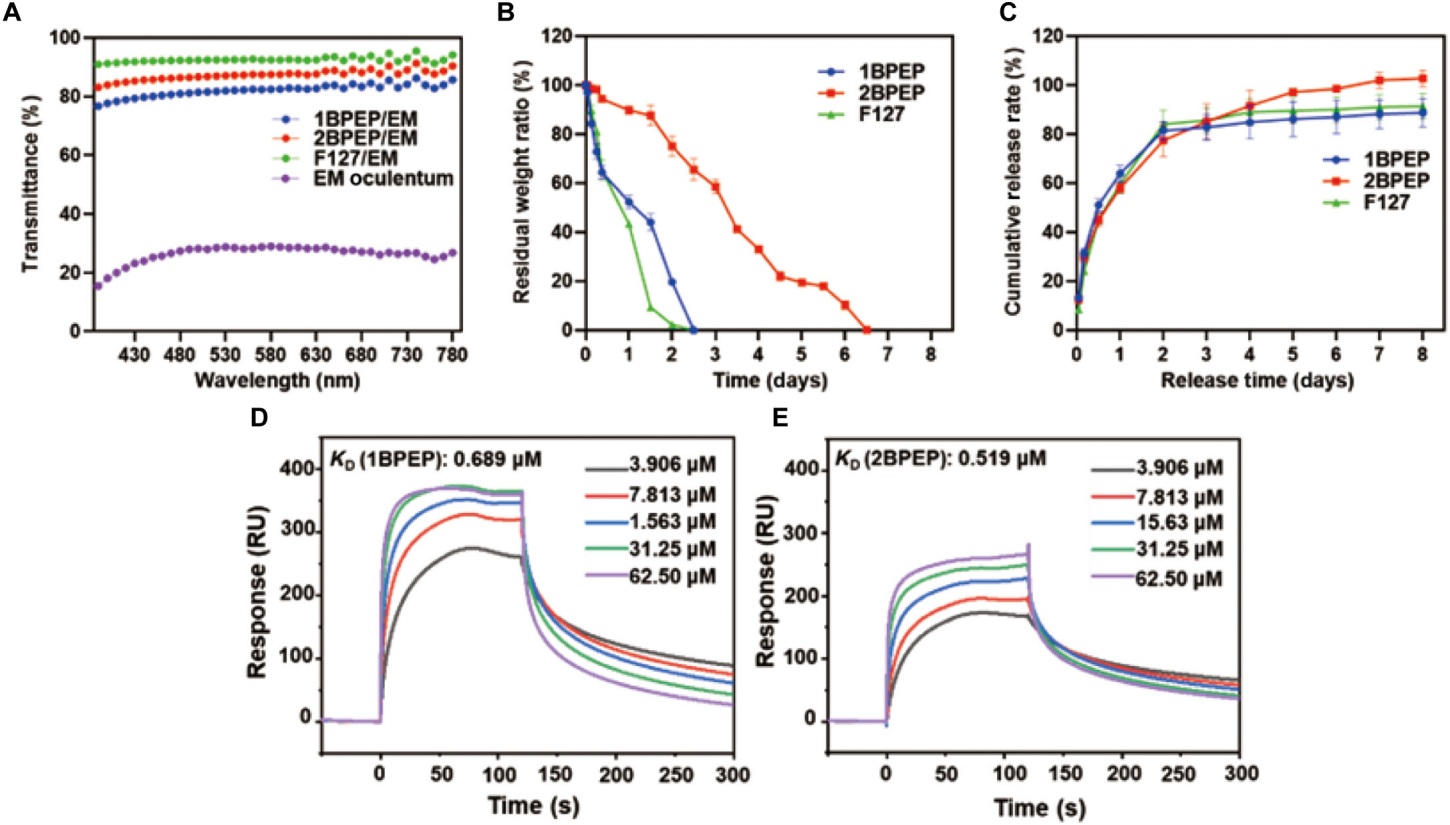
(A) Transmittance of 1B-EM, 2B-EM, and F127-EM hydrogels and EM in the wavelength range of 0 to 780 nm. (B) Residual weight ratio of 1BPEP, 2BPEP, and F127 hydrogels with time. (C) Doxorubicin release curve of 1BPEP, 2BPEP, and F127 hydrogels. Surface plasmon resonance sensograms of (D) 1BPEP and (E) 2BPEP (3.906 to 62.50 μM) binding to mucin in mouse cornea.

1BPEP-EM and 2BPEP-EM hydrogels loaded with amphiphilic 1BPEP and 2BPEP polymers exhibited a light transmittance higher than 70% compared to EM oculentum that exhibited a poor light transmittance. It is worth mentioning that the light transmittance of 1BPEP-EM and 2BPEP-EM hydrogels was close to that of the F127-EM hydrogel (Fig. 4A). In addition, we created 1BPEP, 2BPEP, and F127 hydrogels with EM and tested their transparency. Figure S3 shows that 1BPEP-EM, 2BPEP-EM, and F127-EM exhibited a higher transparency when compared to the EM solution, and showed similar transparency to that of the carbomer gel. The results indicated that BPEP polymer could improve the solubility of EM.

Figure S4 showed the SEM morphology of the 2BPEP and 2BPEP-EM hydrogels. The SEM images revealed that the 2BPEP and 2BPEP-EM hydrogels exhibit an uneven porous network structure, which are likely to contribute to their breathability and facilitate prolonged drug release.

### Evaluation of degradation studies and sustained drug release of hydrogels

In order to explore and compare the gelatinous maintenance ability of the hydrogel systems, we characterized the degradation ratios of various hydrogels. It could be seen from Fig. 4B that the degradation rate of the F127 hydrogel was the fastest, and the degradation ratio of 1BPEP was similar to that of F127, both of which completely degraded within 3 days. However, the degradation ratio of 2BPEP was significantly slower than that of 1BPEP, and its gel state was maintained for more than 6 days, which indicates that the adhesive structure formed by 2BPEP polymer with increased BPOSS content was stronger. Thus, the hydrogels can contribute to the sustained release and retention of the drug at the ocular surface site, thereby improving the bioavailability of the drug.

In order to further explore the drug delivery characteristics of hydrogels, cumulative drug release rates of three hydrogels in PBS release medium containing 0.02% Tween 20 were studied. It can be seen from Fig. 4C that 1BPEP-EM, 2BPEP-EM, and F127-EM hydrogels exhibited a sustained drug release. Further, the 2BPEP-EM hydrogel showed the highest cumulative drug release at the end point and could achieve a sustained drug release ability for up to 8 days.

### Comparison of surface plasmon resonance properties of polymers

Mucin is the primary component of the mucus that covers the surface of the eyeball, and mucus can interact with polymers in a variety of ways (e.g., van der Waals forces, electrostatic interactions, and hydrogen bonds). Some researchers have modified the polymer structure to enhance the interaction time between the polymer and the eyeball surface mucin to improve the effect of drugs [56,57]. BPOSS has been shown to achieve adhesion in the eyeball to the anchoring proteins [58,59]. Therefore, we characterized the binding affinity of 1BPEP and 2BPEP polymers to mucin in order to reflect the adhesion properties of polymers in the eyeball. As shown in Fig. 4D and E, the equilibrium dissociation constants (*K*_D_) of 1BPEP and 2BPEP were 0.689 and 0.519 μM, respectively, with small *K*_D_ values, indicating that the binding affinity of 1BPEP and 2BPEP polymers to mucin was greater. The results demonstrate that the BPOSS group modification on the polymer chain enhanced the adhesion to the eyeball, thus extending the treatment time of the drug in the eyeball.

### In vitro biocompatibility of hydrogels

**Fig. 5.**
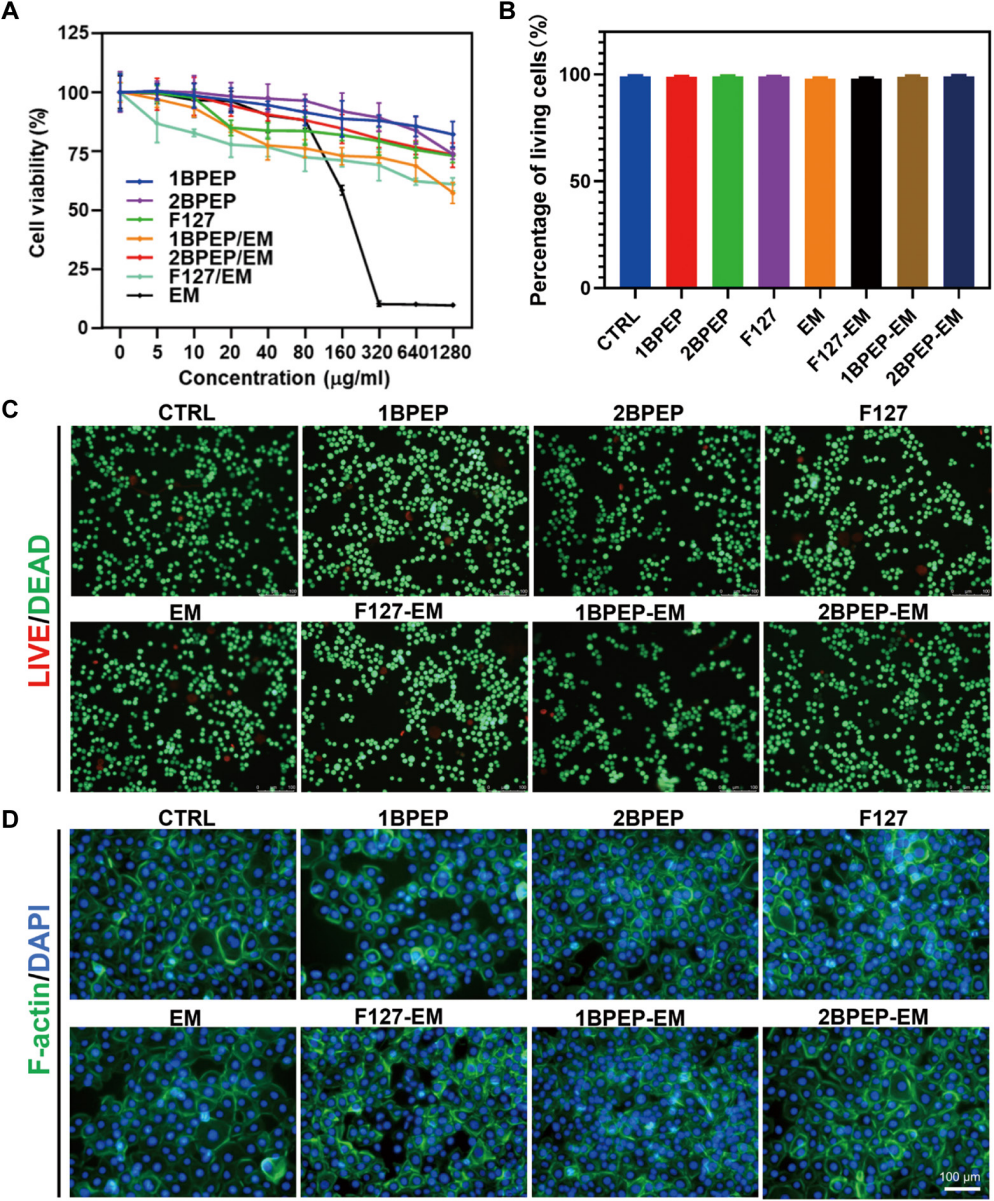
(A) Viability of HCE cells after 24 h of the addition of different formulations using MTT assay. (B) Percentage of living HCE cells cultured in different formulations for 24 h. (C) LIVE/DEAD assay showing HCE cells cultured in different formulations for 24 h. (D) Images of cell morphology (F-actin, green fluorescence) and nucleus (DAPI, blue fluorescence) of HCE cells cultured in different formulations of culture media for 24 h. Data presented as mean ± SEM (*n* = 6).

To assess the biocompatibility of hydrogels, MTT assays were conducted to determine the possible cytotoxic effects on HCE cells. Figure 5A demonstrates that the blank materials F127, 1BPEP, and 2BPEP exhibited no clear toxicological effects on the HCE cells after coculturing for 24 h. However, the EM-loaded materials F127-EM, 1BPEP-EM, 2BPEP-EM, or PBS-EM have impacted the HCE survival. Furthermore, when exposed to EM, the F127 hydrogel was the most hazardous, 1BPEP was less toxic, and 2BPEP was the least toxic. The results indicated that hydrogel preparation could reduce the damage caused by high dose of EM on HCE cells.

In addition, to evaluate the cytocompatibility of hydrogels, we utilized LIVE/DEAD staining, F-actin/DAPI staining, and P63 immunofluorescent staining for visual analysis. After coculturing with the hydrogel for 24 h, LIVE/DEAD staining revealed a predominance of green fluorescent cells across all groups, with negligible presence of dead cells, as shown in Fig. 5B. Figure 5C demonstrates that with the exception of the EM, the ratio of viable cells to total cells in the remaining groups was similar to that of the control group, showing no significant differences. In contrast, the EM solution group displayed a marginally lower proportion of viable cells. Figure 5D reveals that the cells cultured in F127, 1BPEP, 2BPEP, F127-EM, 1BPEP-EM, and 2BPEP-EM did not differ significantly from the control group in terms of cell morphology, adhesion, and spreading. The morphology of cells cultivated in PBS-EM, on the other hand, changed slightly. It suggests that the EM enclosed in the BPOSS hydrogel was less cytotoxic to HCE cells and that the hydrogel formulation can lessen the harm that EM causes to cells. The cell proliferation was assessed by P63 immunofluorescent staining. Figure S5 demonstrates that there are no significant differences between the groups and the BPOSS hydrogel did not exhibit any negative influence on the cell development.

We then performed long-term cytotoxicity experiments to confirm the biocompatibility of the hydrogels. The cells still showed excellent growth orientation when cocultured with F127, 1BPEP, 2BPEP, F127-EM, 1BPEP-EM, and 2BPEP-EM and could grow well within 72 h without inhibition (Fig. S6). Furthermore, cocultures with hydrogels were incubated for 48 and 72 h prior to staining. LIVE/DEAD staining revealed a substantial population of viable cells, comparable to that of the control group. The F-actin/DAPI staining outcomes demonstrated that, after hydrogel treatment, the cells preserved normal morphology akin to that of the control, as illustrated in Figs. S7 and S8. The results demonstrate that the hydrogels maintain superior cytocompatibility, even after extended coculturing with HCE cells.

### Retention of hydrogels on the ocular surface

A hydrogel intended for ocular drug delivery should be an adhesive and ideally prolong drug delivery to the ocular surface. Normal eye drops are quickly cleared out after blinking, but the hydrogel can resist this clearing. Slit lamp photography was used to assess the in situ thermally reversible crosslinking of hydrogels on the ocular surface [60,61]. According to Fig. S9, the EM solution disappeared rapidly from the cornea after blinking. F127-EM, 1BPEP-EM, and 2BPEP-EM hydrogels were able to stay on the cornea for a longer time. However, F127-EM showed some detachment after blinking, while 1BPEP-EM and 2BPEP-EM hydrogels could still keep their integrity after blinking, showing their superior adhesion.

### In vitro antibacterial activity

**Fig. 6.**
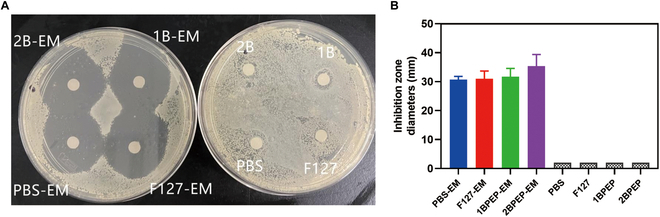
(A) and (B) show the size of the inhibition zone after treatment with various formulations. Data presented as mean ± SEM (*n* = 3).

Building on previous research that validated the antibacterial efficacy of EM-loaded nanoparticles against *S. aureus* using the inhibition zone method, our study employed the same technique to assess the suppressive impact of hydrogels on this bacterium [62]. Figure 6A and B reveals that F127-EM, 1BPEP-EM, 2BPEP-EM, and PBS-EM produced significant inhibition zones in the growth of *S. aureus*, but F127, 1BPEP, 2BPEP, and PBS did not show any inhibition zones. Consequently, the antibacterial efficacy is primarily ascribed to EM, while the blank hydrogel exhibited no antibacterial action against *S. aureus*. Furthermore, compared to F127-EM and PBS-EM, 2BPEP-EM exhibited a larger inhibitory zone, followed by 1BPEP-EM. This could be due to the excellent solubility of EM by 2BPEP.

### In vivo antibacterial test

**Fig. 7.**
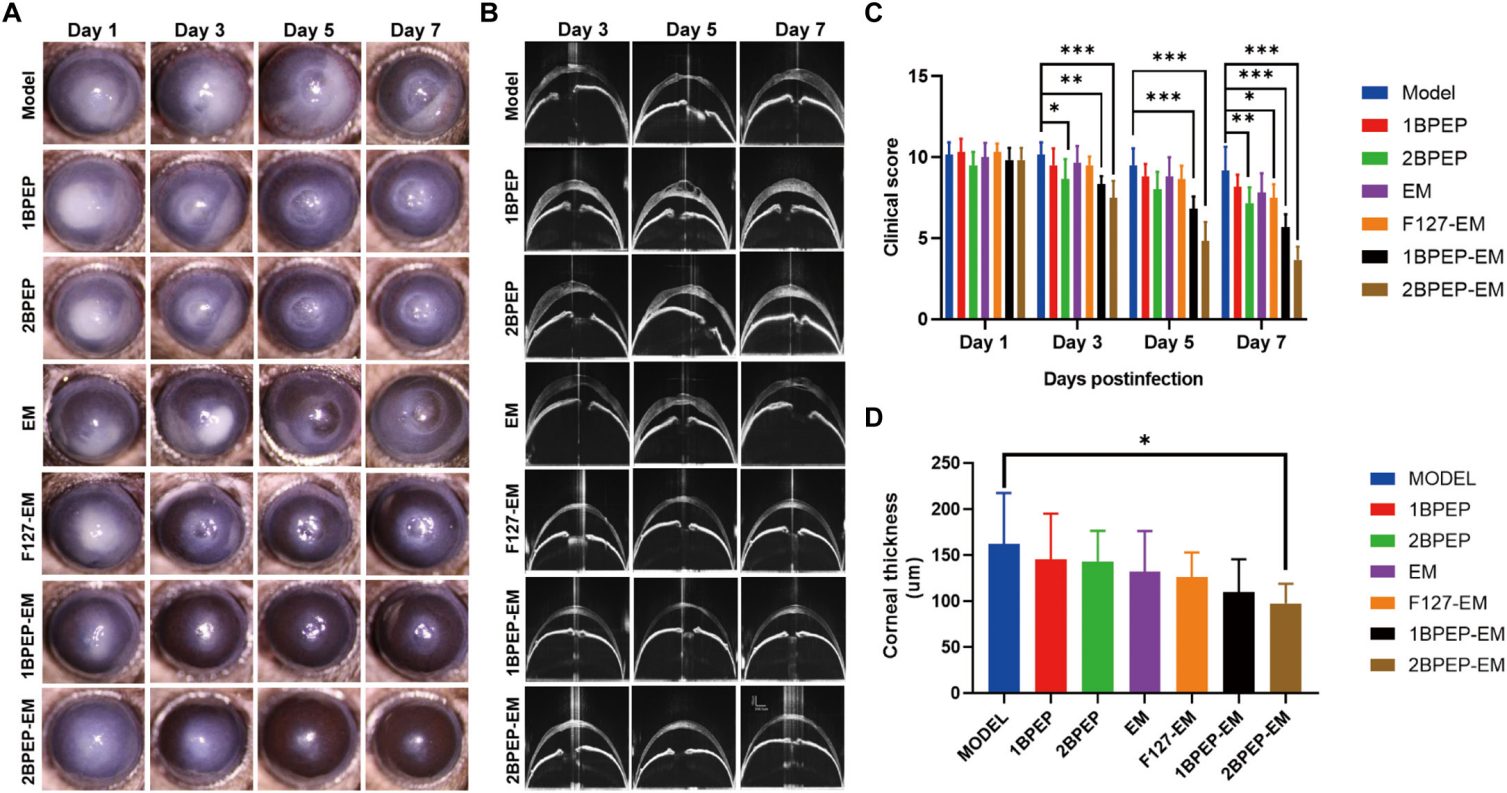
In vivo evaluation of different formulations for bacterial keratitis. (A) Slit lamp images of eyes in each group on days 1, 3, 5, and 7. (B) OCT images of eyes in each group on days 3, 5, and 7. (C) Clinical score for different groups. (D) Quantitative analysis of corneal thickness on day 7. Data presented as mean ± SEM (*n* = 6). *P < 0.05, **P < 0.01, ***P < 0.001.

To further investigate the therapeutic effects of the hydrogel eye drop, *S. aureus* was injected into the mouse corneal stroma to establish an animal model of BK, and the clinical score was calculated using the mouse BK visual scoring method (Table 1). As shown in Fig. 7A, severe corneal opacity and corneal ulcers occurred in each group on the first day after the establishment of the mouse model. On the third day, F127-EM, 1BPEP-EM, and 2BPEP-EM demonstrated varying degrees of remission. The extent of corneal ulcers in 1BPEP-EM and 2BPEP-EM were significantly reduced on the fifth and seventh days, and clinical scores were much lower than in the other groups (Fig. 7C). Remarkably, 2BPEP-EM exhibited the lowest clinical score across all phases, suggesting that it possesses superior therapeutic efficacy.

Optical coherence tomography (OCT) provides a noncontact fast imaging of the cornea, allowing the degree of corneal edema to be quantified, and has been widely used in the infiltration of infectious keratitis [63]. On the third day, all groups developed corneal edema and epithelial irregularities, as illustrated in Fig. 7B. Cornea of the 2BPEP-EM group had nearly regained their initial thickness and regularity on the 5th and 7th day. An improvement in the corneal edema of 1BPEP-EM and F127-EM groups was seen, but remained slightly irregular. In contrast, the ocular edema of MODEL, 1BPEP, 2BPEP, and EM groups worsened rapidly and stayed irregular until the seventh day. The corneal thickness of the 2BPEP-EM group has reverted to normal levels, yet the cornea of the MODEL group is still edematous, according to data on corneal thickness from day 7, as shown in Fig. 7D. The results demonstrated that 2BPEP-EM rapidly alleviates corneal edema and reinstates normalcy in cases of BK, markedly surpassing the performance of the PBS-EM group.

**Fig. 8.**
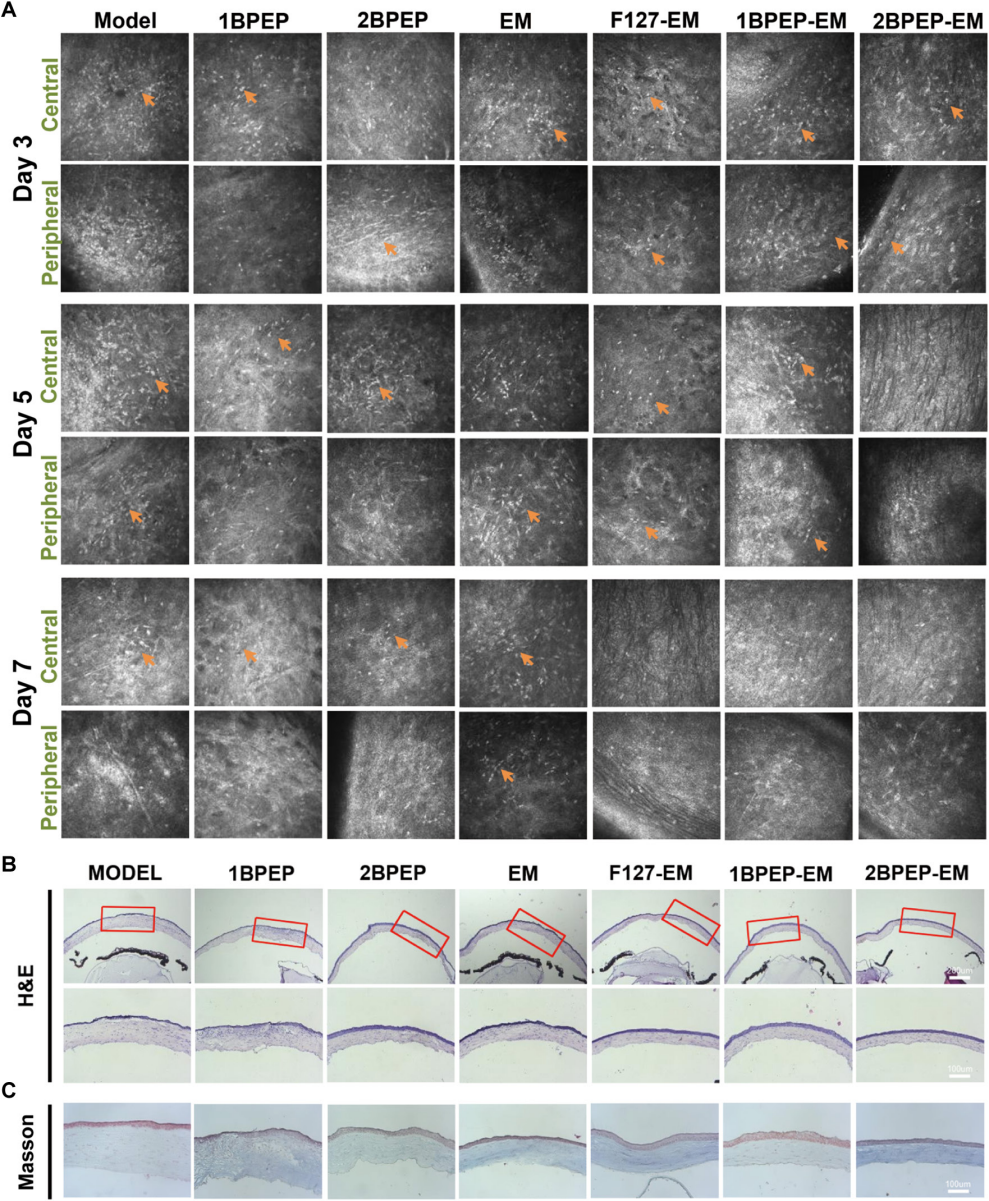
(A) In vivo confocal microscopy images of the central and peripheral corneal stroma in eyes from separate groups on days 3, 5, and 7. Inflammatory cells are represented with orange arrows. (B) H&E staining and (C) Masson staining of different groups of corneal tissue sections.

In vivo confocal microscopy (IVCM) detects the pathological changes in the cornea at the cellular level and conducts a real-time imaging, thus making it a popular tool for diagnosing infectious keratitis [64,65]. As shown in Fig. 8A, each group showed a significant quantity of inflammatory cell infiltration and corneal stroma opacity on the third day after the animal model was established. The inflammatory cells in the 2BPEP-EM group were almost eliminated on the fifth day after the administration of eye drops. The inflammatory cells in the 1BPEP-EM and F127-EM groups were cleared up by the seventh day, and the corneal ulcers healed. However, the MODEL groups, 1BPEP, 2BPEP, and PBS-EM, showed the presence of many inflammatory cells. Overall, 2BPEP-EM had a maximal therapeutic effect in treating BK, significantly higher than EM solution. Therefore, the POSS hydrogel can greatly prolong the retention of EM on the ocular surface and increase its antibacterial activity.

### Histological analysis

H&E staining was used to assess the roughness of corneal epithelium, and Masson staining was used to characterize the recovery of corneal structures. It can be seen in Fig. 8B that the 2BPEP-EM treatment expedited the restoration of corneal epithelial morphology within 7 days, achieving greater normalization of corneal thickness compared to the other groups. Masson staining (Fig. 8C) revealed edema and severe disruption of collagen fibers in the MODEL, 1BPEP, 2BPEP, EM, F127-EM, and 1BPEP-EM treatment groups. However, the corneal structure of the 2BPEP-EM-treated group was almost normal.

**Fig. 9.**
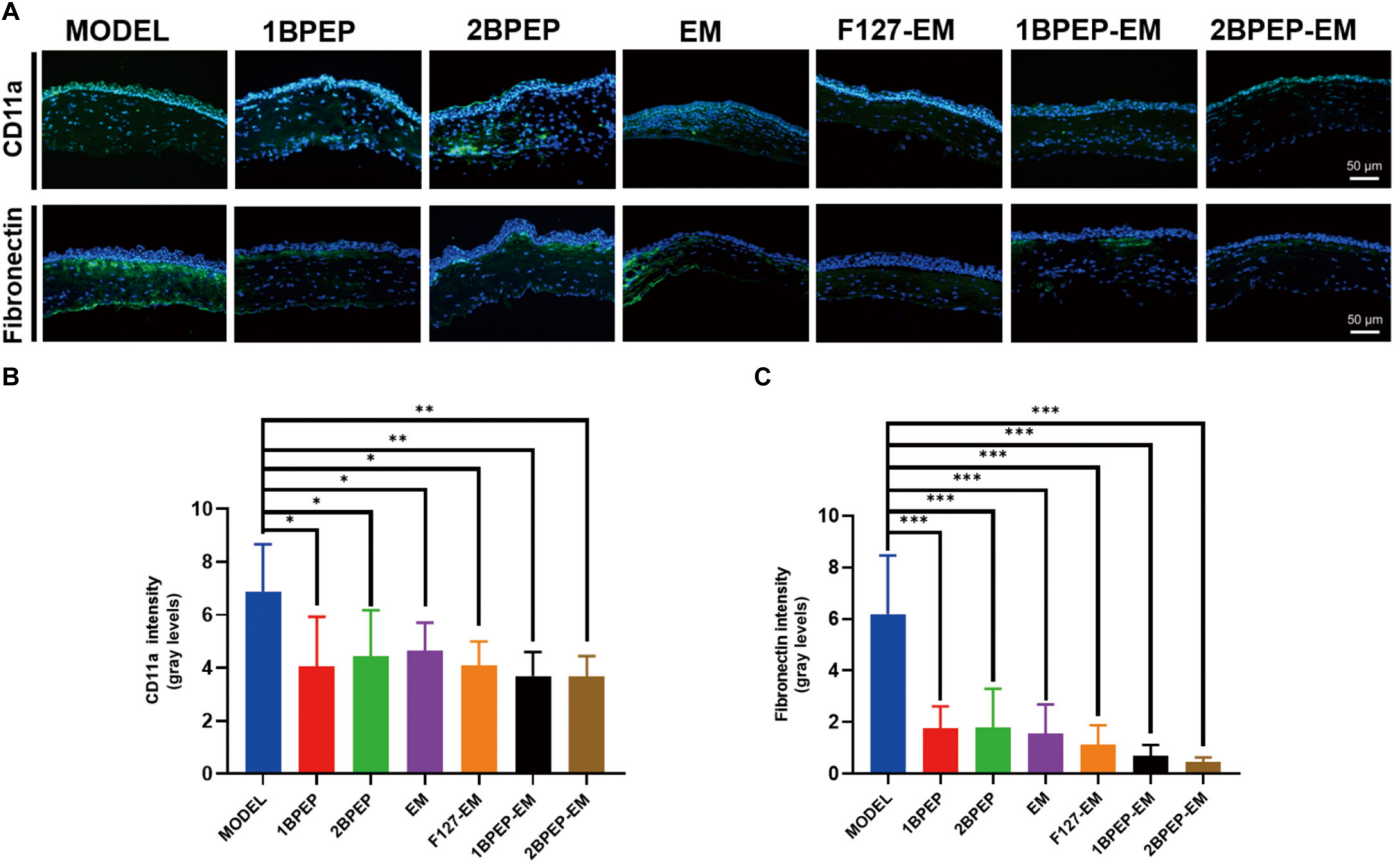
(A) Immunofluorescence staining of fibronectin and CD11a (green) in corneas from different groups. Nuclei were stained with DAPI (blue). Quantitative analysis of (B) fibronectin and (C) CD11a intensity. Data presented as mean ± SEM (*n* = 5 to 6). *P < 0.05, **P < 0.01, ***P < 0.001.

CD11a is a type of inflammatory cell marker that can be used to assess inflammation. The lowered fluorescence intensity of F127-EM, 1BPEP-EM, 2BPEP-EM, and EM was seen by CD11a immunofluorescence (Fig. 9A), demonstrating that EM inhibits inflammation by killing bacteria. Quantitative studies (Fig. 9B) showed that 2BPEP-EM significantly reduced the number of inflammatory cells. Fibronectin is an extracellular structural protein that is used to detect corneal fibrosis. Corneal infections can cause corneal fibrosis, which can lead to corneal opacity and significantly impairs vision. Fibronectin immunofluorescence labeling studies showed that the fluorescence intensity of F127-EM, 1BPEP-EM, and 2BPEP-EM was dramatically decreased compared with other groups, and the anti-fibrosis effect of 2BPEP-EM group was more obvious (Fig. 9A and C). We speculate that EM could kill the bacteria in the early stage, could inhibit inflammation, and exhibited an anti-fibrosis role. Nevertheless, 2BPEP-EM increased the retention time of EM on the ocular surface, so anti-fibrosis was more obvious. In conclusion, 2BPEP-EM promotes the anti-inflammatory and anti-fibrotic effects by increasing the EM retention duration at the ocular surface.

### Safety analysis

The safety of the thermosensitive hydrogel eye drops is indeed indispensable, so we tested the safety of the copolymers by applying topical eye drops to healthy mice for 7 or 20 days. OCT, fundus imaging, and fundus fluorescence angiography were primarily employed in the assessment. As previously stated, OCT is a noncontact method for imaging the cornea and the retina, and is an effective tool for ophthalmic monitoring. Fundus imaging is widely used for screening and diagnosing ocular diseases, as it can show important features such as the optic disc, optic cup, macula, and blood vessels. Furthermore, certain observations concerning hemorrhage, infiltration, and so forth can also be made [66]. The blood vessels of the eye can be seen via fundus fluorescence angiography.

**Fig. 10.**
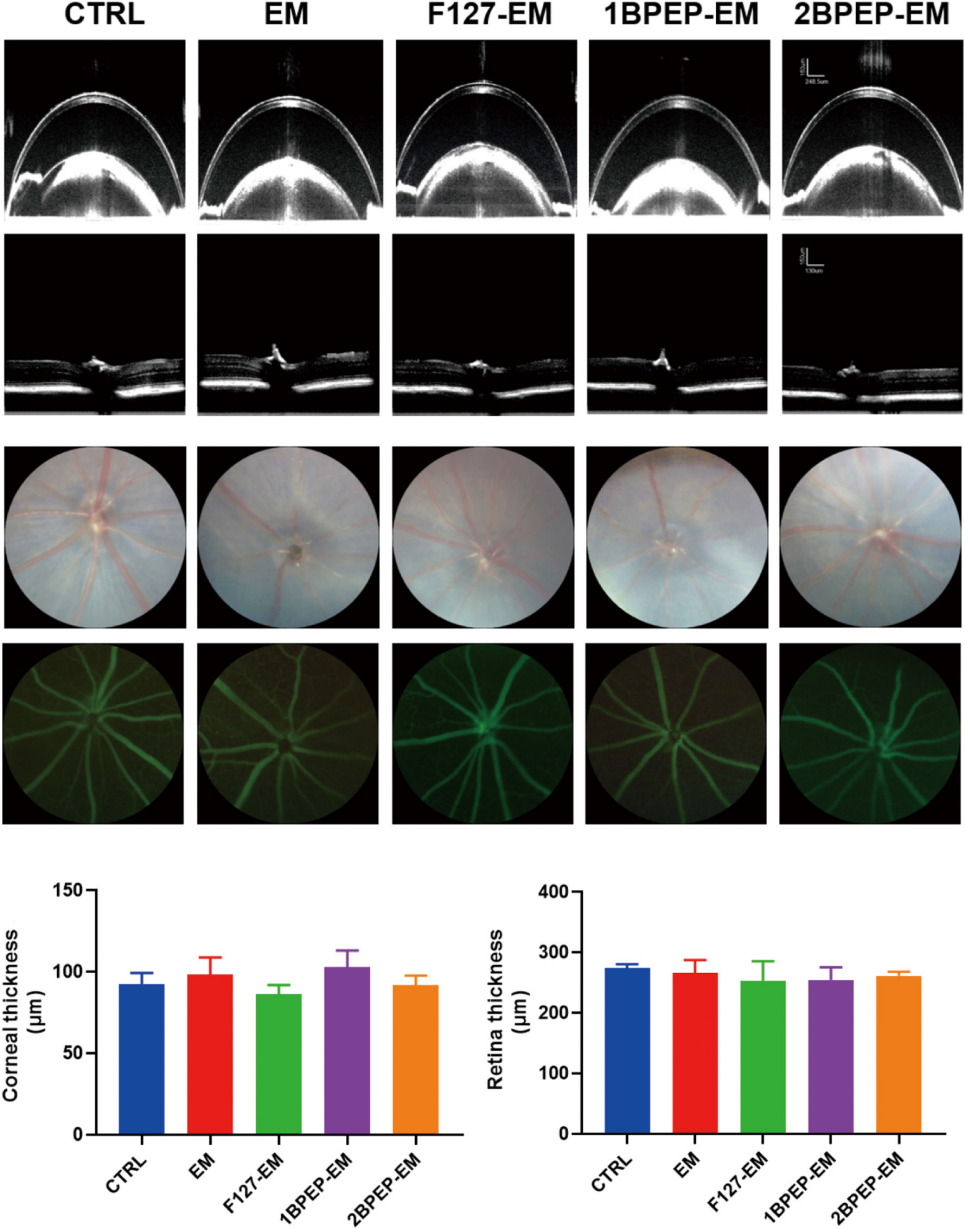
Safety analysis of the formulations. (A) OCT images (cornea and retina) of eyes from different groups. (B) Fundus images from different groups. (C) Corneal thickness and (D) retina thickness did not differ between groups. Data presented as mean ± SEM (*n* = 5).

It was found that at 7 days after application of the hydrogel eye drops, OCT confirmed the regular structure of cornea, absence of significant inflammation, and no discernible leakage in the anterior chamber or narrowing of the atrioventricular space. Posterior segment OCT (PS-OCT) scans indicated that the retinal structure was consistent with that of normal mice, with well-ordered retinal layers and no evidence of detachment or degeneration, as shown in Fig. 10A. Measurement data revealed no significant difference in the thickness of both the cornea and retina compared to the control group, as detailed in Fig. 10C and D, respectively. Fundus imaging and fundus fluorescence angiography presented in Fig. 10B depicted a normal fundus with no signs of retinal leakage, vasodilation, or anomalous pathways, with stable optic disc and retinal vessel morphology, devoid of pigment irregularities, retinal holes, or other alterations.

Furthermore, we conducted long-term evaluations of various hydrogels to investigate their ocular safety. Notably, OCT performed after 20 days of eye drop application revealed that the retinal and corneal layers maintained their normal structure after treatment, with no significant alteration in thickness (Fig. S10A). Additionally, fundus imaging and fundus fluorescence angiography did not reveal any lesions, maintaining consistency with the control group (Fig. S10B). The results showed that the hydrogel has no side effects on the ocular surface, presented no risk even after long-term administration, and exhibited an excellent biosafety profile.

## Conclusion

In this study, we developed a novel PEG-PPG-BPOSS hydrogel containing EM for treating BK. This hydrogel exhibited excellent physical characteristics and could self-assemble and gel on the ocular surface. Its gelation prolonged the retention time of EM on the eye surface, and this hydrogel improved the water solubility of EM. Furthermore, the developed PEG-PPG-BPOSS hydrogel exhibited a superior biocompatibility and bioavailability, as evident from the in vitro and in vivo studies. More crucially, we found that this thermosensitive hydrogel eye drops showed a stronger therapeutic effect for BK than EM solution. As a result, the BPOSS thermosensitive hydrogel offers a more comprehensive concept in the therapeutic situation and has a promising future in the treatment of ocular illnesses.

## Ethical Approval

This study does not involve human participants, human data, or human tissue. This study involved experimental animals. The Xiamen University Experimental Animal Ethics Committee gave its approval to the research protocol. The Association for Research in Vision and Ophthalmology’s Statement for the Use of Animals in Ophthalmic and Vision Research was followed when using animals.

## Data Availability

The datasets used and/or analyzed during the current study are available from the corresponding author on reasonable request.
