# Supplementary material for: Thermosensitive Polyhedral Oligomeric Silsesquioxane Hybrid Hydrogel Enhances the Antibacterial Efficiency of Erythromycin in Bacterial Keratitis
Source: Biomater Res. 2024 Jul 22;28:0033. doi: 10.34133/bmr.0033 (PMC11260774; doi:10.34133/bmr.0033)
Supplement: Supplementary 1 — Figs. S1 to S10 Table S1 [file bmr.0033.f1.docx]

**Thermosensitive polyhedral oligomeric silsesquioxane hybrid hydrogel enhances the antibacterial efficiency of erythromycin in bacterial keratitis**

Lan Zheng ^a, 1^, Ying Chen ^b, 1^, Yi Han ^a,d^, Jingwei Lin ^a^, Kai Fan ^b^, Mengyuan Wang ^a^, Ting Teng ^d^, Xiuqin Yang ^b^, Lingjie Ke ^b^, Muyuan Li ^f^, Shujia Guo ^a^, Zibiao Li ^e,^ *, Yunlong Wu ^b,^ *, Cheng Li ^a,c,d,^ *

^a^ Fujian Provincial Key Laboratory of Ophthalmology and Visual Science & Ocular Surface and Corneal Diseases, Eye Institute & Affiliated Xiamen Eye Center, School of Medicine, Xiamen University, Xiamen 361102, PR China

^b^ Fujian Provincial Key Laboratory of Innovative Drug Target Research and State Key Laboratory of Cellular Stress Biology, School of Pharmaceutical Sciences, Xiamen University, Xiamen 361102, PR China

^c^ Huaxia Eye Hospital of Quanzhou, Quanzhou, Fujian 362000, China

^d^ Department of Ophthalmology, The First Affiliated Hospital of University of South China, Hengyang Medical School, University of South China, Hengyang, Hunan, 421001, PR China

^e^ Institute of Materials Research and Engineering, A*STAR (Agency for Science, Technology and Research), Singapore 138634, Singapore

^f^ Shandong First Medical University & Shandong Academy of Medical Sciences, Jinan, 250117, Shandong Province, China

**Supporting Information**

**Supporting Table**

**Table S1.** Mean particle size of BPEP micelles in aqueous solution

| temperature / °C | 0.5BPEP / nm^a^ | | | 1BPEP / nm^a^ | | | 2BPEP / nm^a^ | | |
| --- | --- | --- | --- | --- | --- | --- | --- | --- | --- |
|  | 0.1 wt% | 0.5 wt% | 1 wt% | 0.1 wt% | 0.5 wt% | 1 wt% | 0.1 wt% | 0.5 wt% | 1 wt% |
| 25 | 49.79 | 49.72 | 53.96 | 55.39 | 51.42 | 58.64 | 58.87 | 58.43 | 69.62 |
| 37 | 42.61 | 42.39 | 43.80 | 48.02 | 41.39 | 44.44 | 52.23 | 51.72 | 61.57 |
| 70 | 38.2 | 53.36 | 65.00 | 40.02 | 55.13 | 68.31 | 51.76 | 80.18 | 84.74 |

^a^ Mean diameter (by intensity) of particles at different concentration measured by DLS.

**Supporting Figures**


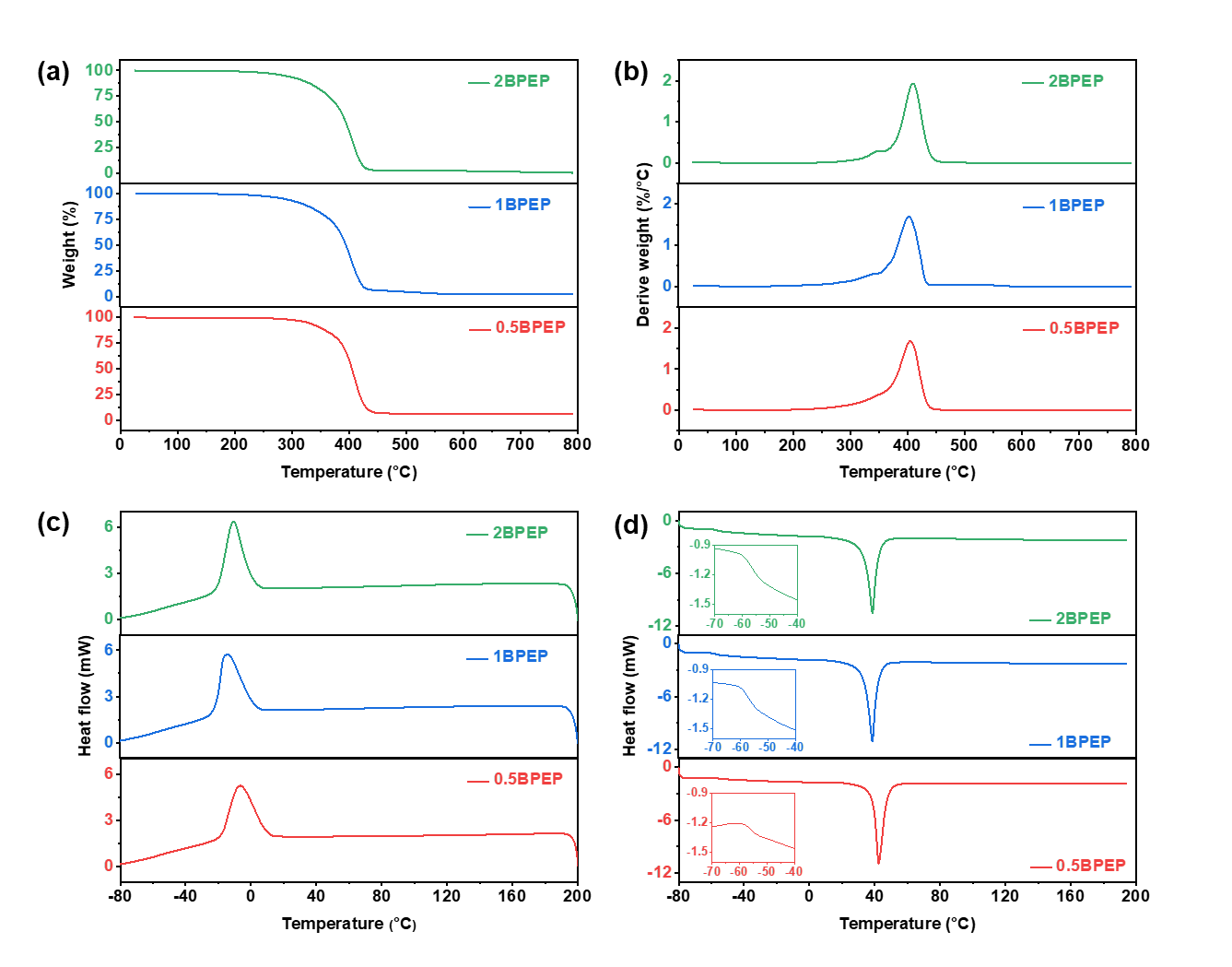


**Figure S1.** (a) Weight curves and (b) derive weight curves of nBPEP measured by TGA. (c) Cooling curves and (d) heating curves of nBPEP measured by DSC. The inserted graphs in (d) indicate the process of glass transition.


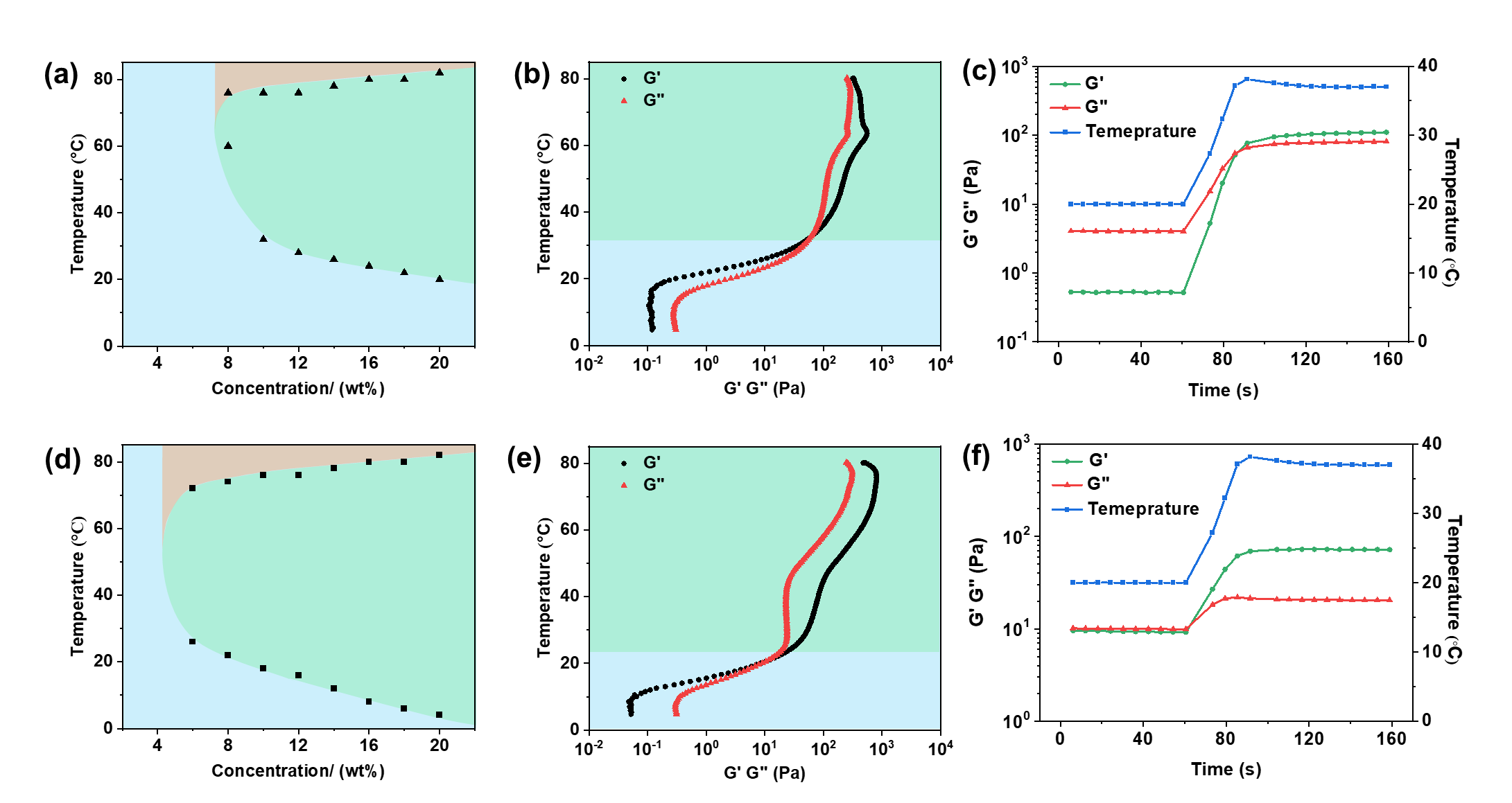


**Figure S2.** Phase diagram of (a) 0.5BPEP and (b) 2BPEP determined by tube inverting method. Rheological properties of (c) 0.5BPEP (10 wt%) and (d) 2BPEP (6 wt%) samples in temperature sweep. Rheological properties of (e) 0.5BPEP (10 wt%) and (f) 2BPEP (6 wt%) samples in temperature ramp.


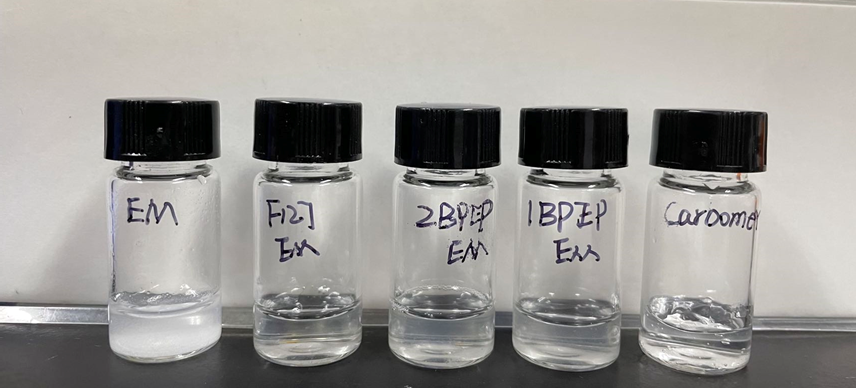


**Figure S3.** Transparency observation of Commercial carbomer and erythromycin, F127-EM, 1BPEP-EM, 2BPEP-EM formulations.

**
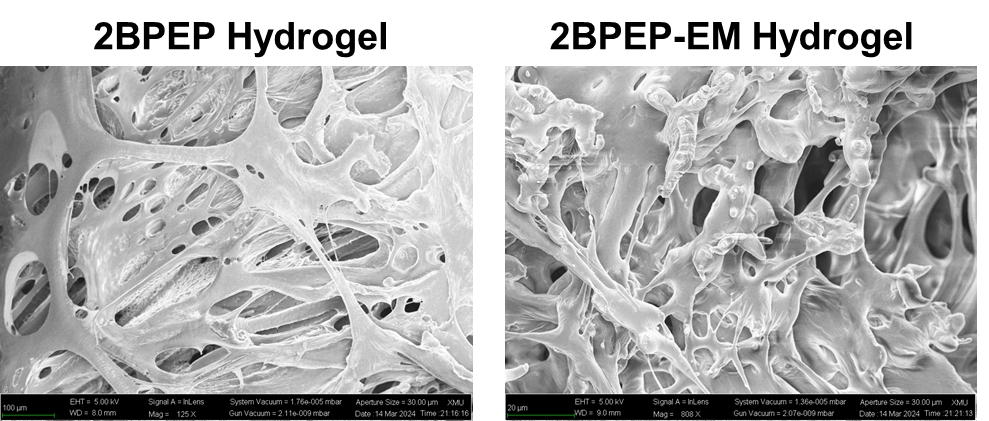
**

**Figure S4.** Scanning electron microscope images of the 2BPEP hydrogel and 2BPEP-EM hydrogel.


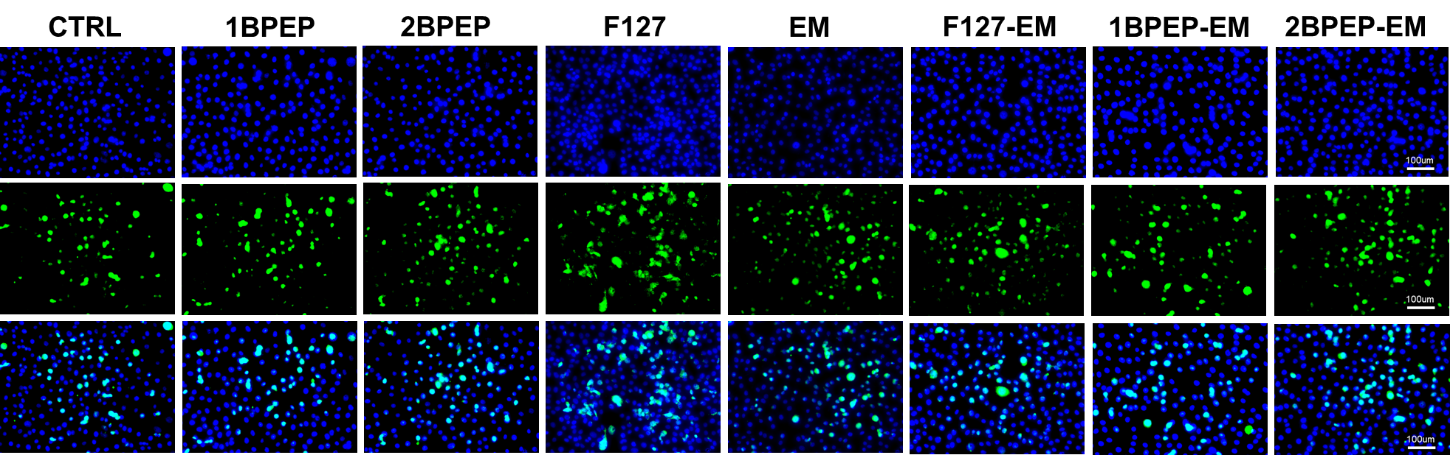


**Figure S5.** P63 (green fluorescence) and DAPI (blue fluorescence) of HCE cells cultured in medium containing different formulation for 24 hours.


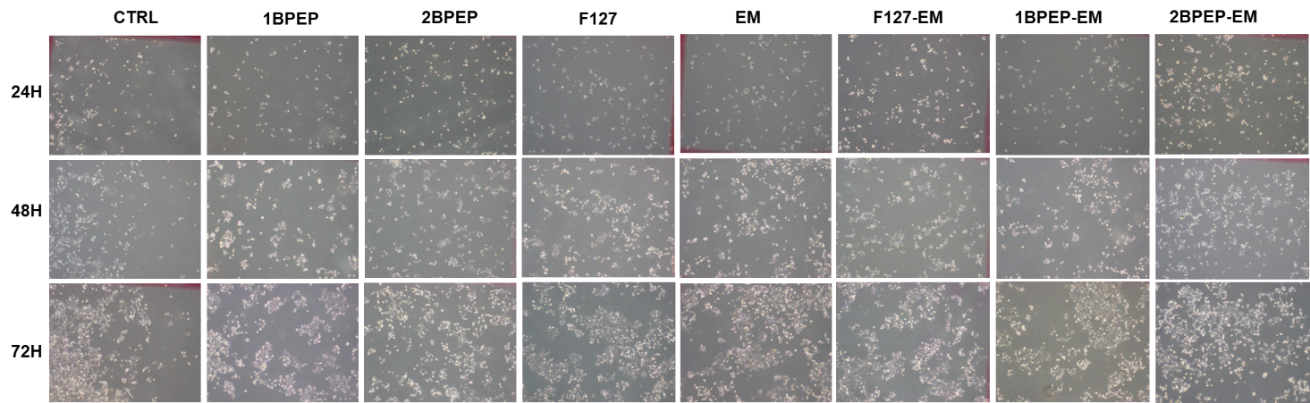


**Figure S6.** HCE cells were co-cultured with different groups of formulations for 72 hours.


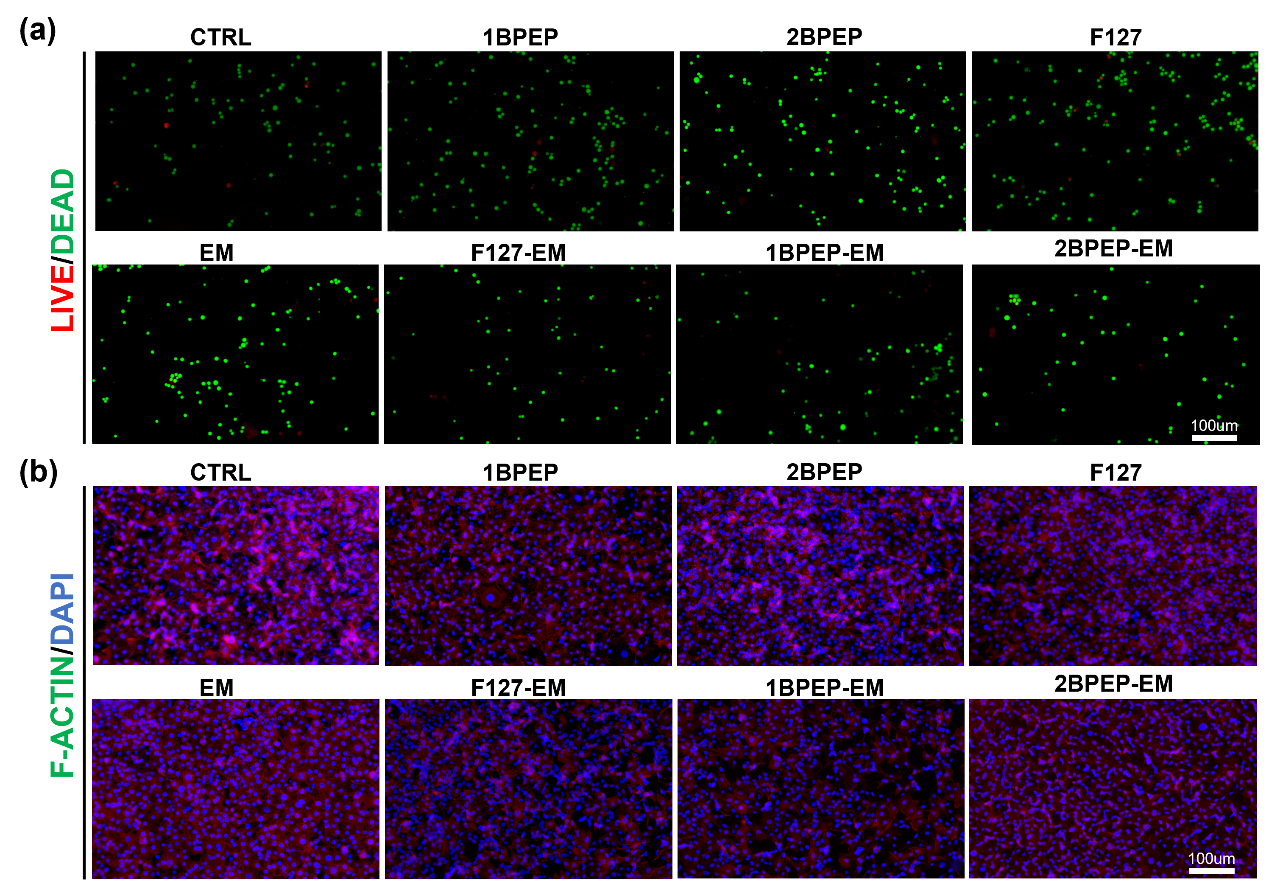


**Figure S7.** (a) LIVE/DEAD assay showing HCE cells cultured in different formulations for 48 hours. (b) Images of cell morphology (F-actin, red fluorescence) and nucleus (DAPI, blue fluorescence) of HCE cells cultured in different formulations of culture media for 48 hours.


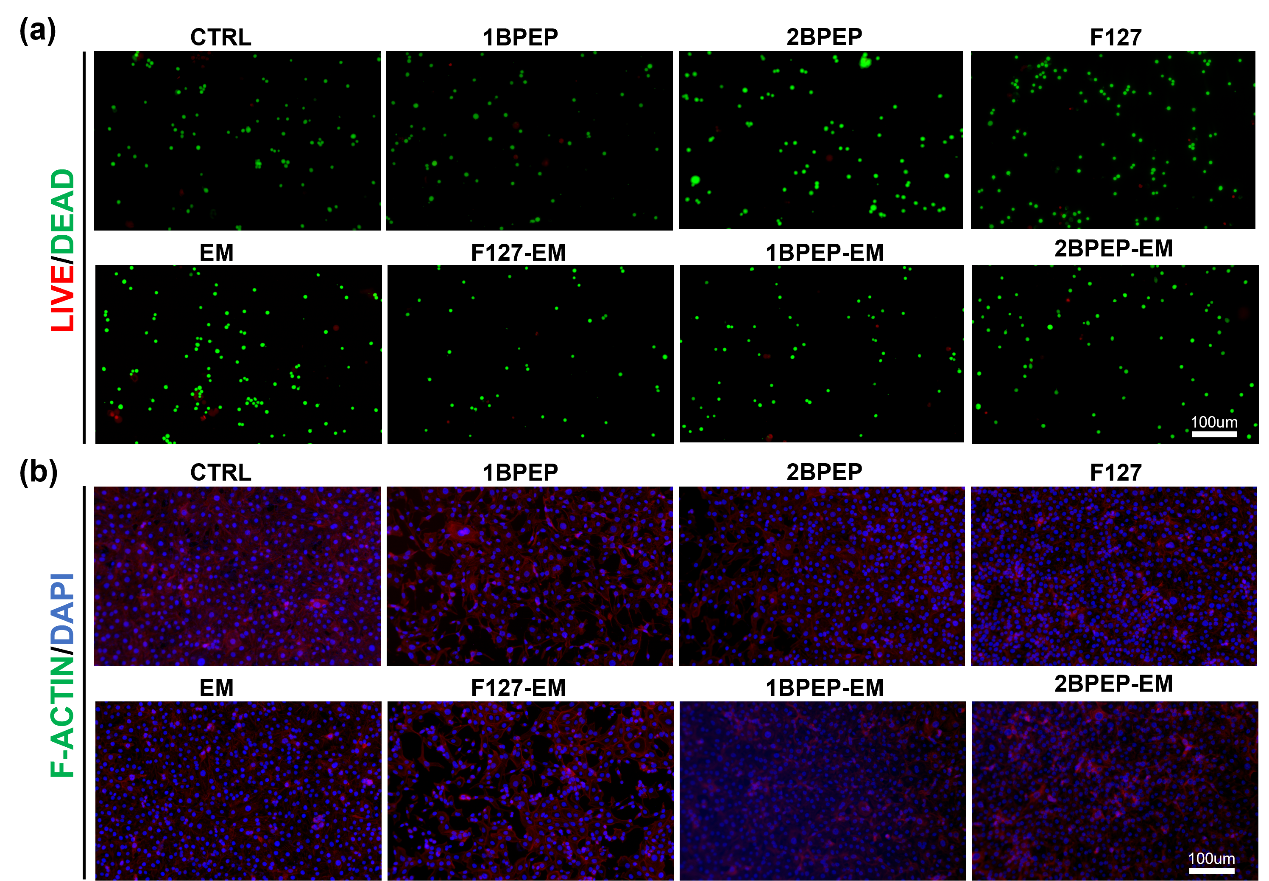


**Figure S8.** (a) LIVE/DEAD assay showing HCE cells cultured in different formulations for 72 hours. (b) Images of cell morphology (F-actin, red fluorescence) and nucleus (DAPI, blue fluorescence) of HCE cells cultured in different formulations of culture media for 72 hours.


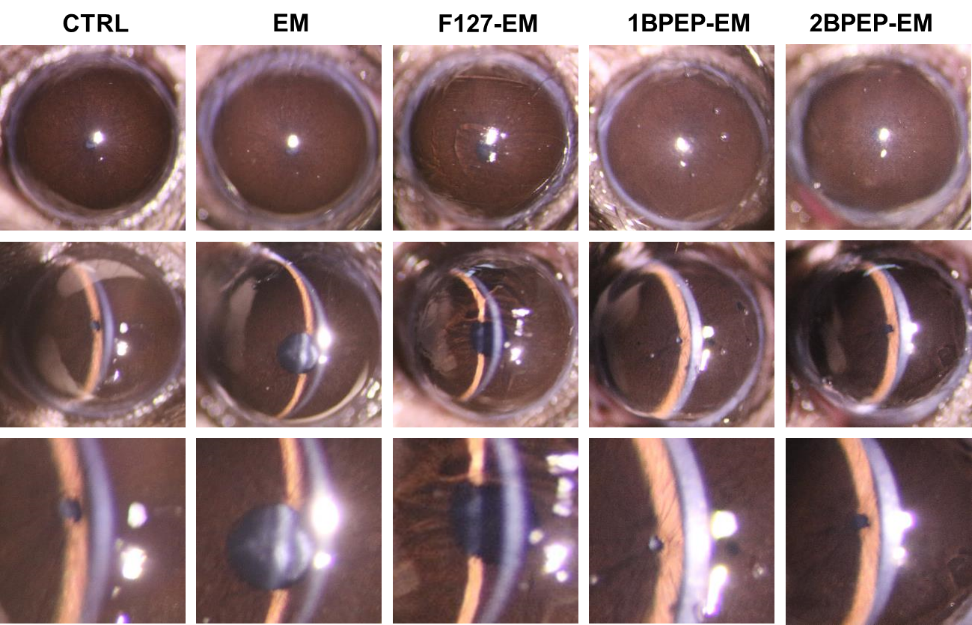


**Figure S9.** Slit lamp images of different groups of hydrogels at the ocular surface after blinking.


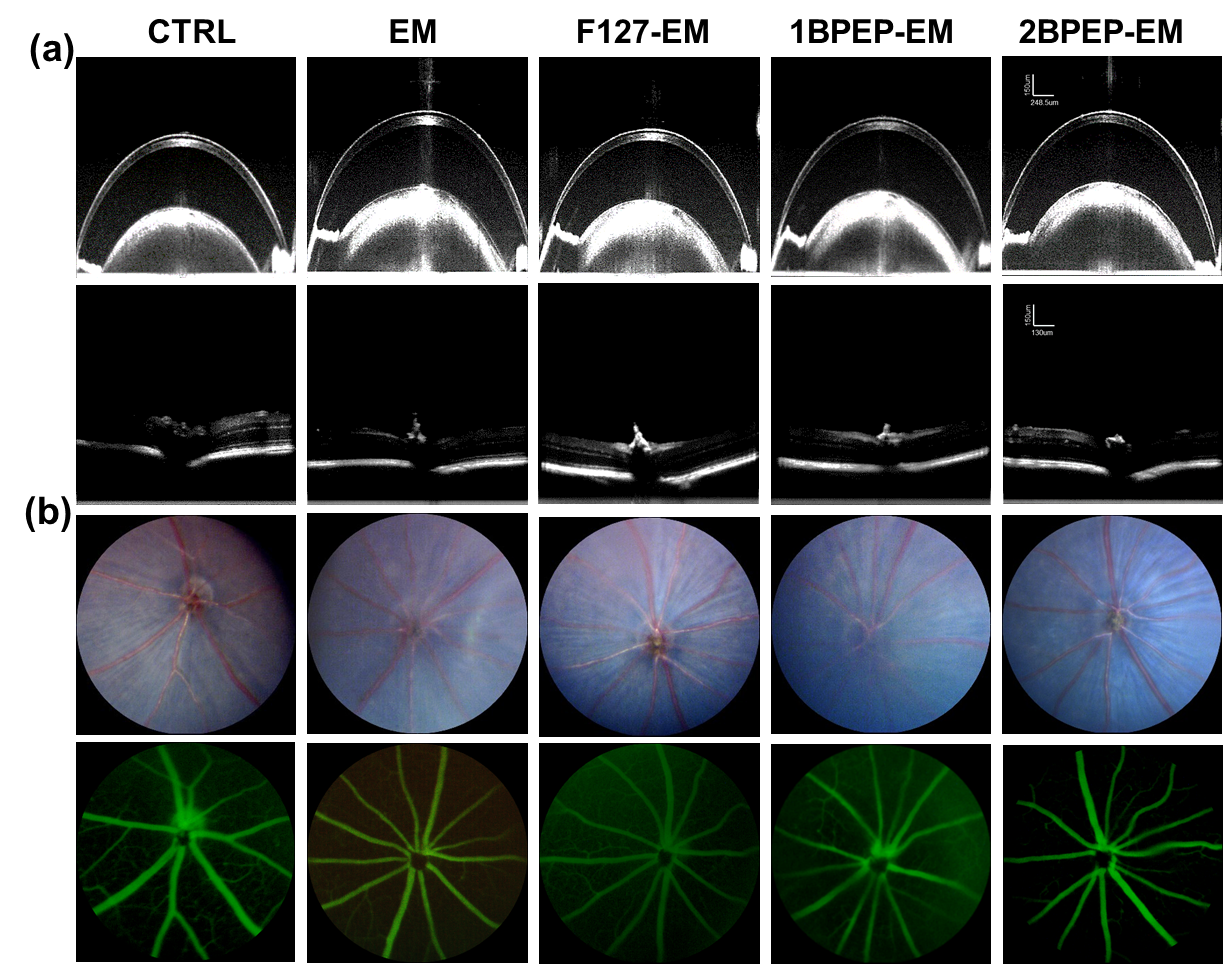


**Figure S10.** Long-term safety evaluation. OCT images(a) and fundus images(b) of different groups of materials after 20 days of drug spotting.
